# Functional loss of p53 cooperates with the *in vivo* microenvironment to promote malignant progression of gastric cancers

**DOI:** 10.1038/s41598-018-20572-1

**Published:** 2018-02-02

**Authors:** Junko Ohtsuka, Hiroko Oshima, Issei Ezawa, Ryo Abe, Masanobu Oshima, Rieko Ohki

**Affiliations:** 1 Laboratory of Fundamental Oncology, National Cancer Center Research Institute, Tsukiji 5-1-1, Chuo-ku, Tokyo, 104-0045 Japan; 2 Division of Immunobiology, Research Institute for Biomedical Sciences, Tokyo University of Science, 2669 Yamazaki, Noda City, Chiba, 278-0022 Japan; 3 Division of Genetics, Cancer Research Institute, Kanazawa University, Kanazawa, 920-1192 Japan; 4 Graduate School of Advanced Science and Engineering, Waseda University, 3-4-1 Okubo, Shinjuku-ku, Tokyo, 169-8555 Japan

## Abstract

*p53* mutations are frequently detected in malignant gastric cancers. However, the molecular mechanisms by which loss of p53 function promotes gastric cancer are not clear. We utilized Gan mice (*K19-Wnt1/C2mE*), which have functional p53 and develop intestinal-type gastric tumors, to investigate the role of p53 in gastric cancer progression by knocking out *p53*. We found that gastric epithelial cells acquire tumorigenicity in the subcutis of C57BL/6 mice as a result of Wnt activation, COX-2 activation and p53 deficiency. With repeated allograft transfers, these gastric epithelial cells gradually acquired the properties of malignant gastric cancer. Loss of p53 conferred cell stemness and induced epithelial to mesenchymal transition (EMT) in gastric epithelial cells, and these properties were further enhanced by the *in vivo* microenvironment, ultimately leading to gastric cancer formation and metastasis. We also found that the *in vivo* microenvironment enhanced activation of the COX-2 pathway, which further contributed to cancer progression. With this system, we have succeeded in recapitulating the development of malignant gastric cancer from gastric epithelial cells in a normal immune environment.

## Introduction

Gastric cancer has the second highest mortality among cancers worldwide, and research efforts to clarify the mechanisms underlying the development of this deadly cancer are urgently needed. The tumor suppressor gene *p53* is one of the most frequently mutated genes in human cancers, and *p53* mutations occur in 0 to 77% of stomach cancers^1,2^. Mutation of *p53* has been observed starting at the early stages of gastric cancer and this frequency increases as the malignancy progresses^2^. Most gastric cancers are adenocarcinomas, and gastric adenocarcinomas can be broadly divided into two types: differentiated and undifferentiated types^3^. The majority of gastric cancers are differentiated adenocarcinomas, and in general have a relatively low malignancy. In contrast, the undifferentiated types tend to become more malignant and can become highly metastatic^4^. It is also known that the loss of E-cadherin expression in gastric cancers correlates with cellular dedifferentiation and glandular disintegration^5^. Furthermore, chronic Helicobacter pylori infection is known to be involved in the development of gastric cancer^6^. These observations suggest that functional loss of p53, acquisition of an undifferentiated phenotype, and an inflammatory response are essential for the development of malignant gastric cancer.

*K19-Wnt1/C2mE* mice, commonly called Gan mice, are a transgenic mouse line that develops intestinal-type gastric tumor due to activation of the Wnt and PGE_2_ pathways^7^. Activation of the Wnt pathway is found in more than 30% of human gastric cancers, and contributes to the self-renewal of cancer stem cells^8^. It has also been reported that gastric epithelial cells in Gan mice acquire the ability to self-renew as a result of Wnt activation^7^. In addition, activation of the PGE_2_ pathway is also frequently observed in gastric cancers, and this signaling promotes the formation of inflammatory microenvironments involving macrophages and fibroblasts that contribute to gastric cancer development^9,10^. Gastric tumors from Gan mice have a gene expression profile similar to that of human intestinal-type differentiated gastric adenocarcinoma, and the malignancy of the tumor cells is relatively low^11^.

In order to investigate the role of p53 in the formation and malignant progression of gastric cancer, we crossed Gan mice with *p53*-deficient mice to produce a *p53*−/− Gan line. Gastric epithelial cells derived from *p53*−/− Gan mice were tumorigenic when transplanted to immunocompetent C57BL/6 mice, and over time and with repeated allograft *in vivo*, the transplanted gastric epithelial cells acquired the properties of malignant gastric cancer. With this model, we have succeeded in recapitulating the development of malignant gastric cancer from gastric epithelial cells in a normal immune environment.

## Results

### Loss of the tumor suppressor gene *p5*3 enhances the self-renewal ability of gastric epithelial cells and results in an increase in undifferentiated cells in the gastric epithelia

**Figure 1:**
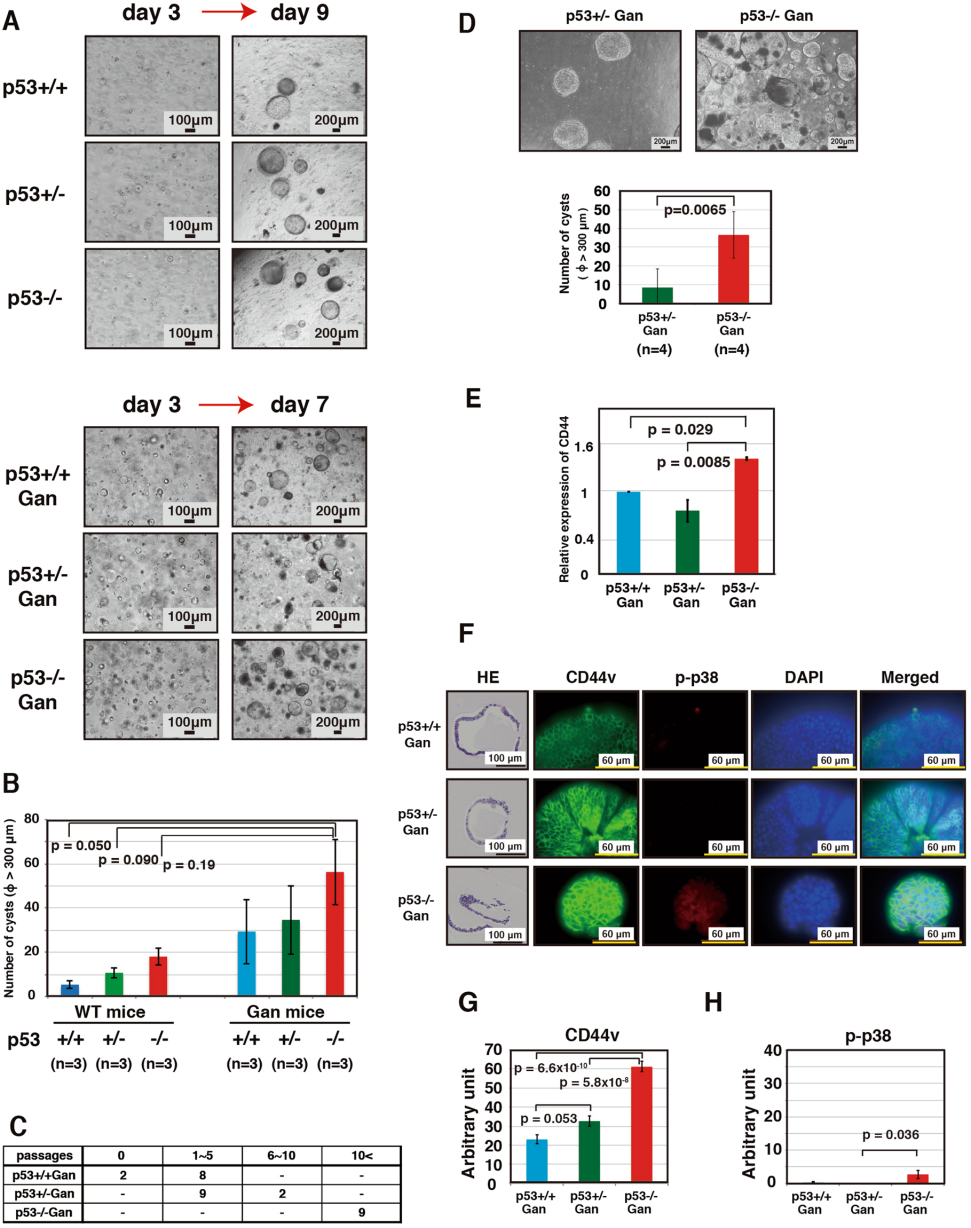
Gastric epithelial cells of *p53*−/− Gan mouse show enhanced proliferative and self-renewal abilities. Gastric epithelial cells from *p53*+/+, *p53*+/−, *p53*−/− mice at 8−11 weeks of age and *p53*+/+ Gan, *p53*+/− Gan, *p53*−/− Gan mice at 7–10 weeks of age were cultured in Matrigel. Cells were seeded at a density of 1.3 × 10^5^ cells per 3−cm dish on day 0 and analyzed on day 7~9. (**A**,**B**) The numbers of cystic structures derived from *p53*−/− gastric epithelial cells are increased in mice of both wild-type and the Gan background compared to the respective *p53*+/+ cells. Representative photographs of the primary cultured gastric epithelial cells in Matrigel from *p53*+/+, *p53*+/− and *p53*−/− mice (upper panels) on days 3 and 9 and *p53*+/+ Gan, *p53*+/−Gan and *p53*−/− Gan mice (lower panels) on days 3 and 7 are shown (**A**). The mean number of cystic structures>300 mm in diameter in Matrigel were counted and shown as a graph (**B**). Indicated numbers (n) of mice were analyzed. (**C**) The numbers of passages for the organoids derived from the indicated mice are shown. The culture period for one passage was for 6 days. Organoid cells from *p53*−/− Gan mice continued to proliferate, while cells from *p53*+/+ Gan mice ceased to proliferate after several passages. (**D**) After one passage, gastric epithelial cells from *p53*+/− Gan and *p53*−/− Gan mice were cultured in Matrigel. The mean number of cystic structures>300 mm in diameter in Matrigel were counted as shown in graph. Indicated numbers (n) of mice were analyzed. (**E**) Expression levels of *CD44* were analyzed by real-time PCR. Expression of *CD44* was enhanced in *p53*−/− Gan organoids. (**F**–**H**) Organoids were cultured in Matrigel on cover glass, and immunostaining was performed using the indicated antibodies. The quantitative analysis of fluorescent immunostaining was analyzed using ImageJ (**G**,**H**).

Although mutation or deletion of *p53* is frequently observed in stomach cancer, the detailed molecular mechanisms by which loss of p53 promotes gastric cancer has not been elucidated. To address this issue, we crossed Gan (*K19-Wnt1/C2mE* transgenic) mice, a gastric cancer mouse model, and *p53*-deficient mice to generate *p53*+/+ Gan, *p53*+/− Gan and *p53*−/− Gan mice. We next compared the properties of the gastric epithelial cells derived from each of these mice in a primary epithelial organoid culture system using Matrigel. In addition, we examined gastric epithelial cells derived from *p53*+/+, *p53*+/− and *p53*−/− mice. As shown in Fig. S1A, all organoids formed round cystic structures. We analyzed mRNA expression of *p53*, together with several p53 target genes (*Mdm2*, *p21*, *PHLDA3*, *FUCA1*, *IER5*, *PLK2*, *RPRM*, *PMAIP1* and *PTP4A1*), in organoids derived from *p5*3+/+ Gan, *p53*+/− Gan, and *p53*−/− Gan mice^12–20^. Expression levels of *p53* were lower in the *p53* heterozygous, and still lower in the homozygous deletion organoids (Fig. S1B). We have analyzed the expression of 9 p53 target genes (*Mdm2*, *p21*, *PHLDA3*, *FUCA1*, *IER5*, *PLK2*, *RPRM*, *PMAIP1* and *PTP4A1*, Fig. S1C–K). Expression of *Mdm2*, *PHLDA3* and *FUCA1* were significantly decreased in the *p53*−/− Gan organoids (Fig. S1C,E and F), and loss of one allele of p53 only partially affected the expression of p53 target genes. As shown in Fig. 1A and B, gastric epithelial cells from *p53*−/− mice tended to produce more cysts during cultivation compared with gastric epithelial cells from *p53*+/+ or *p53*+/− mice in both the wild-type and Gan mice background. There were overall more cysts in the Gan mice compared to the non-Gan mice (Fig. 1A and B). We also found that while epithelial cells from *p53*+/+ Gan and *p53*+/− Gan ceased to proliferate after several passages, cells derived from *p53*−/− Gan continued to proliferate and could be passaged for long periods (Fig. 1C). In addition, after one passage, the ability of cells derived from *p53*−/− Gan to form cysts was significantly higher than with *p53*+/− Gan cells (Fig. 1D). We also have demonstrated that abilities of *p53*−/− *C2mE* and *p53*−/− *Wnt1* gastric epithelial cells to form cysts were lower than for *p53*−/− Gan gastric epithelial cells, showing that the cyst-forming ability of *p53*−/− Gan gastric epithelial cells depends on both C2mE and Wnt1 activity (Fig. S2). Furthermore, expression of *CD44* mRNA, a stem cell marker, was markedly elevated in *p53*−/− Gan epithelial cells, indicating an increase in the number of undifferentiated cells in the gastric epithelia (Fig. 1E). On the other hand, expression of CD44 in *p53*+/− Gan was similar to *p53*+/+ Gan under the conditions utilized. Expression of CD44v, an isoform of CD44 particularly associated with tumorigenesis, was also increased due to loss of p53 (Fig. 1F and G). It has been reported that increased expression of CD44v results in decreased p38 activation^21^, and we therefore analyzed phospho-p38, an indicator of p38 activation. Contrary to our expectation, phospho-p38 levels were slightly increased in *p53*−/− Gan organoids (Fig. 1F and H). The above results show that p53 deficiency, as well as Wnt activity and COX-2 activity, enhances the self-renewal ability of gastric epithelial cells and results in an increased number of undifferentiated cells in the gastric epithelia.

### Gastric epithelial cells of *p53*−/− Gan mice acquire tumorigenicity

**Figure 2:**
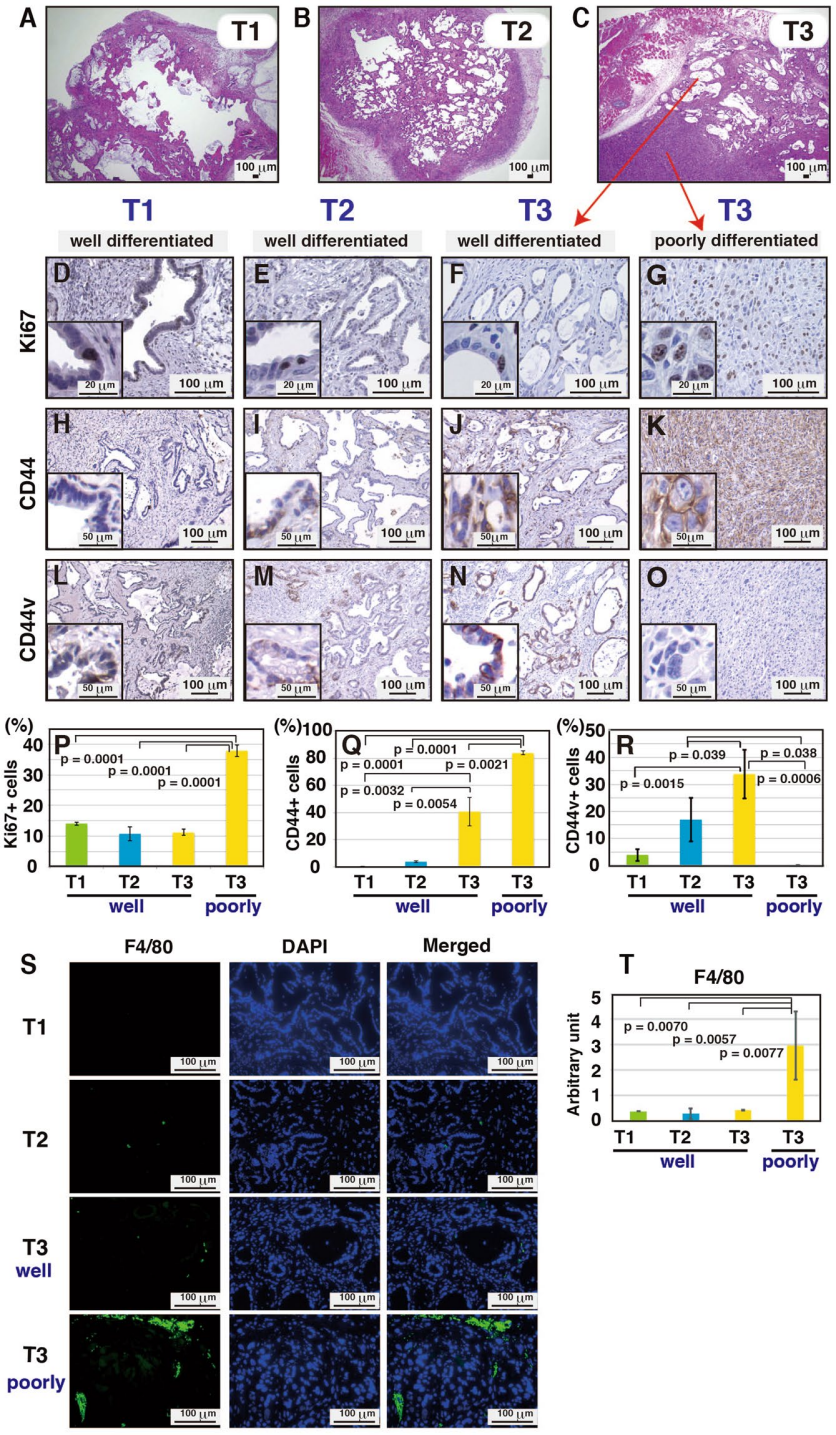
Gastric epithelial cells of *p53*−/− Gan mouse acquire tumorigenicity. (**A**–**C**) Organoids derived from *p53*−/− Gan mice were passaged in C57BL/6 mice repeatedly and histological examination was performed by hematoxylin and eosin (H&E) staining. After 3 passages in mice, a poorly and well-differentiated mixed-type tumor was observed. (**D**–**O**) T1-T3 cells were stained with anti-Ki67, anti-CD44 or anti-CD44v antibodies. Representative photos are shown. (**P**–**R**) The ratios of Ki67 (**P**), CD44 (**Q**) and CD44v (**R**) -positive cells from T1, T2 and T3 tumors were measured from 3 microscopic fields (1 field of Ki67 or CD44v is 0.5 mm^2^ and 1 field of CD44 is 0.2 mm^2^) and shown in graphs. (**S**,**T**) Immunostaining for macrophage marker F4/80. For each tumor, 3 samples were stained, and fluorescent immunostaining was quantified using ImageJ. Macrophage infiltration was increased in the poorly differentiated type tumor from T3 cells.

Cells derived from the gastric epithelium of *p53*+/+, *p53*+/−, *p53*−/− and *p53*+/+ Gan mice did not show any tumor-forming potential when subcutaneously injected into C57BL/6 mice (Table 1). In addition, epithelial cells derived from *p53*+/− Gan mice formed few tumors. In contrast, cells derived from *p53*−/− Gan mice formed tumors at a 100% frequency. Thus, gastric epithelial cells acquire tumorigenicity in the subcutis of C57BL/6 mice due to p53 deficiency along with Wnt and COX-2 activation reported to be involved in tumor formation in Gan mice. We repeatedly inoculated tumorigenic epithelial cells derived from *p53*−/− Gan into the subcutis of C57BL/6 mice, and histologically examined the resulting tumors at each passage. The tumors formed from primary epithelial cells (T1), and after one passage (T2) were differentiated-type adenocarcinomas (Fig. 2A and B). In contrast, tumors that had been passaged twice (T3) were a mixture of well-differentiated and poorly-differentiated types of adenocarcinomas (Fig. 2C). We found that Ki67 positivity was similar among the differentiated type tumors in T1, T2 and T3, but was remarkably high in the poorly-differentiated type tumor areas of T3 (Fig. 2D–G, and P). In addition, CD44 positivity was dramatically higher in the differentiated tumor areas of T3 compared to T1 and T2, and it was still higher in poorly-differentiated tumor areas of T3 (Fig. 2H–K, and Q). Interestingly, we also found that while CD44v positivity was dramatically higher in the differentiated tumor areas of T3 compared to T1 and T2, very little CD44v positivity was observed in poorly-differentiated tumor areas of T3 (Fig. 2L–O, and R). Furthermore, when the tumors were stained with a macrophage marker, macrophage infiltration was induced significantly in the poorly-differentiated tumor areas of T3, while very little macrophage infiltration was observed in the differentiated type tumors in T1, T2 and T3 (Fig. 2S and T). This suggests that the increase in total CD44 associated with decrease in CD44v and invasion of immune cells may be related to tumor morphological changes.

**Table 1:** Gastric epithelial cells from *p53*−/− Gan mice acquire tumorigenicity, and cells derived from T3 acquire a highly malignant phenotype. Organoids derived from various genotypes were analyzed for tumorigenicity and metastatic potential using C57BL/6 mice. Cells used for analysis were gastric epithelial cells from 7–10 week old mice, stomach cancer cells formed in 33–48 week old mice, T3-3D cells and T3-2D cells. Cells were implanted into the subcutis, spleen, stomach and peritoneal cavity. The number of transplants (injections), and the numbers of tumors formed in each organ are shown in the table. Tumorigenicity was defined as tumor growth to 5 mm or more. Donor mice: mice that provided the cells used for transplantation. Recipient mice: mice that received the transplant. *Where there was a significant decrease in tumor formation frequency between C57BL/6 (Table 1) and BALB/c-nu/nu mice (Table 2). ^#^Where there was a decrease in frequency but this did not reach a statistically relevant difference.

Site	Cells	Donor mice	Recipient mice	Injections	Tumors	Liver	Lung	Diaphragm	Spleen	Colon & Pancreas	Stomach
Subcutis	**p53+/+**	7	10	11	0	0	0	0	0	0	0
	**p53+/−**	5	5	6	0	0	0	0	0	0	0
	**p53−/−**	5	6	7	0	0	0	0	0	0	0
	**p53+/+ Gan**	4	15	20	0	0	0	0	0	0	0
	**p53+/− Gan**	5	10	12	1	0	0	0	0	0	0
	**p53−/− Gan**	8	15	15	15*	0	0	0	0	0	0
	**p53+/+ Gan tumor**	7	6	6	0	0	0	0	0	0	0
	**p53+/− Gan tumor**	9	9	7	0	0	0	0	0	0	0
	**p53−/− Gan tumor**	7	7	7	7*	0	0	0	0	0	0
	**T3-3D**	5	12	12	12*	0	0	0	0	0	0
	**T3-2D**	1	21	25	25	0	3	0	0	0	0
Spleen	**p53−/−Gan**	3	7	7	0	0	0	0	0	0	0
	**T3-3D**	4	8	8	1	1	0	0	0	0	0
	**T3-2D**	1	9	9	9	8*	3^#^	0	0	0	0
Stomach	**T3-2D**	1	19	19	19	5^#^	0	2^#^	3	0	0
I.P.	**T3-2D**	1	12	12	12	4	0	7	2	9	1

### Change in the tumor morphology from differentiated to undifferentiated type is associated with EMT

**Figure 3:**
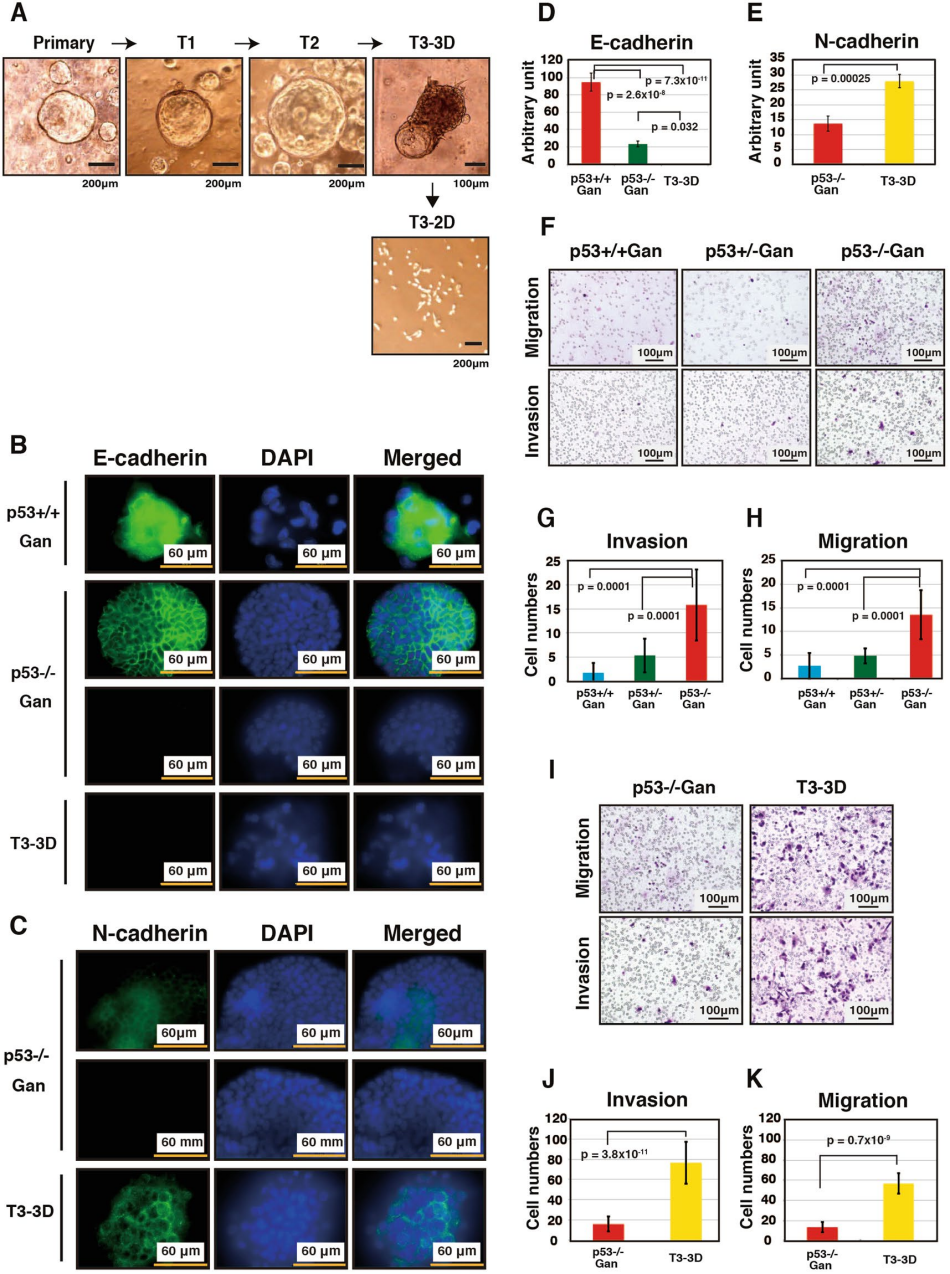
EMT induction and enhanced cell motility in T3 cells. (**A**) Gastric cystic structure in three-dimensional cultivation of *p53*−/− Gan primary cells, T1 and T3 tumor cells were analyzed. Cells were seeded at a density of 1.3 × 10^5^ cells per 3-cm dish on day 0 and cultured to day 6 or to 7. Cells derived from T3 formed cystic structures with protrusions in Matrigel (T3-3D cells). T3 cells can also be cultured in the 2D plate (T3-2D cells). (**B**–**E**) *p53*−/− Gan primary cells and T3 tumor cells were seeded at a density of 1 × 10^5^ cells per 12 well dish on day 0 and immunostained on day 5. (**B**,**C**) Organoids were cultured in Matrigel on cover glass, and immunostaining of E-cadherin (**B**,**D**) and N-cadherin (**C**,**E**) were performed. Fluorescent immunostaining was quantitatively analyzed using ImageJ. (**F**–**K**) Invasion and migration assays were performed using ThinCert inserts. *p53*+/+ Gan, *p53*+/− Gan, *p53*−/− Gan and T3-3D cells (1 × 10^5^ cells) were seeded into the upper insert in serum-free medium. Cell migration was determined using inserts for 12-well multiwell plates with 8.0 µm pore sizes. Cell invasion was analyzed using the same inserts coated with Matrigel. Representative photos are shown (**F**,**I**). The results presented are an average of 8-13 random microscopic fields (**G**,**H**,**J**,**K**).

Cells derived from T1-T3 were introduced into Matrigel three-dimensional cultures. While the T1 and T2 cells formed rounded cysts similar to those observed in primary gastric epithelial cell cultures, T3 cells (T3-3D cells) formed cysts with protrusions (Fig. 3A). In addition, while E-cadherin expression was high in *p53*+/+ Gan organoids, it progressively decreased in *p53*−/− Gan organoids (Fig. 3B and D), and was weak or absent in T3-3D cells. In addition, N-cadherin expression was higher in T3-3D cells compared to *p53*−/− Gan-derived organoids (Figs 3C,E and S3A). In T3-3D cells, we observed decreased E-cadherin and increased N-cadherin expression, suggesting that these cells have undergone EMT. Since EMT is closely related to cell motility, we analyzed the migratory and invasive abilities of these cells. Among the *p53*+/+ Gan, *p53*+/− Gan, *p53*−/− Gan primary gastric epithelial cells, the *p53*−/− Gan cells exhibited remarkably high migration and invasiveness (Fig. 3F–H). Furthermore, when we compared cultures of *p53*−/− Gan primary gastric epithelial cells and T3-3D cells, we observed markedly higher migration and invasiveness in the T3-3D cells (Figs 3I–K and S3B,C). These data collectively show that loss of p53 function in the Gan genetic background initiates EMT, which is further promoted by the *in vivo* microenvironment, culminating in complete EMT and high cell motility in the T3-3D cells.

### Morphological change from a differentiated type to an undifferentiated type is associated with a change in CD44 splicing and enhanced activation of the p38 and PGE_2_ pathways

**Figure 4:**
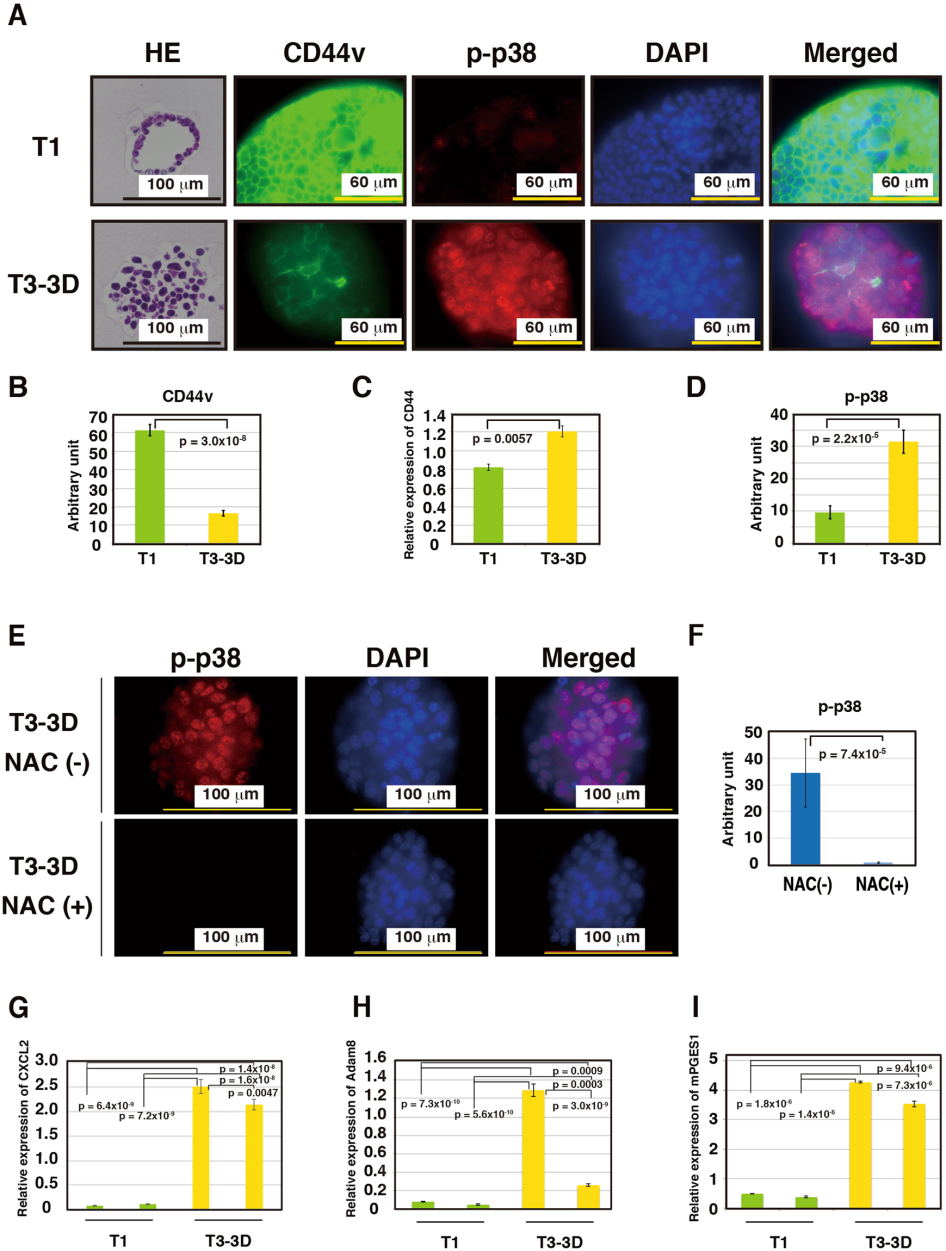
Change in the splicing pattern of CD44, strong activation of p38 and strong activation of COX-2 pathway is observed in T3 cells. Gastric cystic structure in three-dimensional cultivation of *p53*−/− Gan primary cells, T1 and T3 tumor cells were analyzed. (**A**,**B**,**D**) T1 and T3 tumor cells were seeded at a density of 1 × 10^5^ cells per 12-well dish on day 0 and immunostained on day 5. Organoids were cultured in Matrigel on cover glass, and immunostaining was performed. Immunostaining of CD44v (**A**,**B**) and p-p38 (**A**,**D**) were performed. Fluorescent immunostaining was quantitatively analyzed using ImageJ. (**C**) Cells were seeded at a density of 1.3 × 10^5^ cells per 3-cm dish on day 0 and cultured for 6 or 7 days. Expression levels of *CD44* were analyzed by real-time PCR. Expression of *CD44* was slightly enhanced in T3-3D cells compared to T1 cells. (**E**,**F**) T3-3D cells were treated with or without NAC and immunostained for p-p38. Fluorescent immunostaining was quantitatively analyzed using ImageJ. (**G**–**I**) Expression levels of *CXCL2*, *Adam8* and *mPGES-1* were analyzed by real-time PCR. Expression of *CXCL2*, *Adam8* and *mPGES-1* was enhanced in T3 cells compared to T1 cells.

Since we observed a change in the CD44 expression pattern in the T3 tumors (Fig. 2J,K,N and O), we next analyzed CD44 and CD44v expression in T3-3D cells. While total *CD44* mRNA expression levels were slightly elevated, CD44v expression was dramatically reduced in T3-3D cells compared to T1 cells and *p53*−/− Gan gastric epithelial cells (Figs 4A–C and S3D,E). We also found that p38 activation was dramatically increased in T3-3D cells (Figs 4A,D and S3F). It has been reported that reduced expression of CD44v is associated with increased ROS levels and higher p38 activity^21^. Also, activation of p38 has been reported to promote EMT^22^, which was observed in the T3-3D cells. Decreased CD44v may cause the higher p38 activation observed in T3-3D cells, and we therefore treated the T3-3D cells with NAC (N-acetyl-L-cysteine) to inhibit ROS. As shown in Fig. 4E and F, NAC treatment of T3-3D cells resulted in a dramatic decrease in p38 activation, indicating that ROS caused increased p38 activation in T3-3D cells. We also analyzed the mRNA expression of three genes in the PGE_2_ pathway, *CXCL2*, *Adam8* and *mPGES1*^23,24^. As shown in Figs 4G,H and S3G,H, expressions of *CXCL2* and *Adam8* were dramatically increased in T3-3D cells, and may have contributed to the recruitment of macrophages and to the morphological changes observed in T3 tumors. On the other hand, expression of *mPGES1* was high in only a some of the T3-3D cells (Figs 4I and S3I). Taken together, these data show that *in vivo* culture of the *p53*−/− Gan gastric epithelial cells was accompanied by changes in CD44 splicing, high p38 activity and high PGE_2_ pathway activation.

### Establishment of malignant gastric cancer cell line from T3 tumor cells

**Figure 5:**
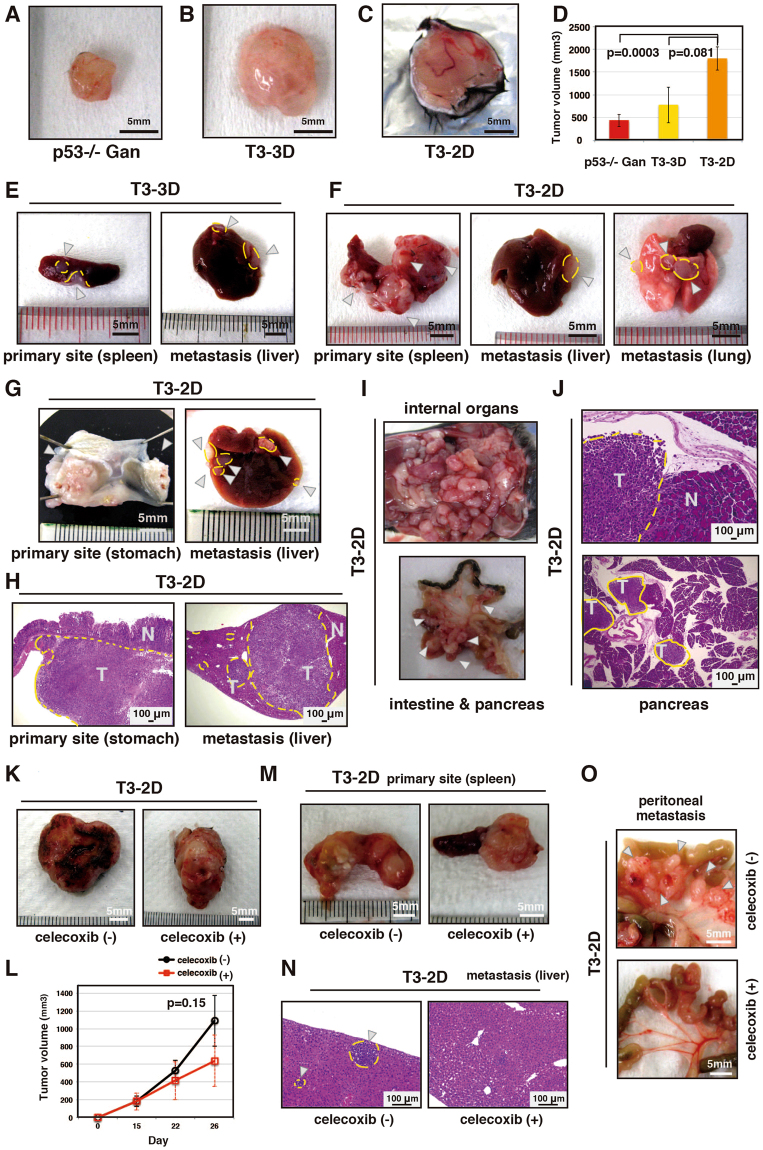
Cells derived from T3 acquire a highly malignant phenotype, and the tumorigenicity and metastatic potential of T3-2D cells are suppressed by an anti-inflammatory drug. (**A**–**C**) Representative images of the tumors formed by subcutaneous implantation. (**D**) Tumor volumes formed by subcutaneous implantation was calculated as (mm^3^) = (length × (width) 2/2). (**E**,**F**) Representative images of the tumors formed by splenic implantation. White arrows indicate tumors. (**G**–**J**) Representative images of the tumors formed by implantation to the stomach (**G**,**H**) and peritoneal cavity (**I**,**J**). Visual observation (**G**,**I**) and (**H** & **E**) staining (**H**,**J**) are shown. White arrows indicate tumors. (**K**) Representative images of the tumors formed by subcutaneous implantation with or without celecoxib treatment. (**L**) Tumor volume formed by subcutaneous implantation with or without celecoxib treatment was calculated as in Fig. 5D. (**M**,**N**) Representative images of the tumors formed by splenic implantation. White arrows indicate tumors. (**O**) Representative images of the tumors formed by implantation to the peritoneal cavity. White arrows indicate tumors.

T3-3D cells could be maintained in two dimensional cultures, and could be subcultured for more than 3 months. From this we inferred that we had established a cell line from the T3 tumor cells, here designated T3-2D. We compared the ability of *p53*−/− Gan gastric epithelial cells, T3-3D and T3-2D cells to form grafts and to metastasize following implantation into the subcutis and spleen (Table 1). As shown in Fig. 5A–D, subcutaneously implanted tumors from T3-3D grew slightly larger than those from *p53*−/− Gan gastric epithelial cells, and tumors from T3-2D grew still larger. It was also found that T3-2D can metastasize to the lungs, albeit infrequently (Table 1). After splenic implantation, T3-2D cells were frequently found engrafted in the liver, while liver engraftment of T3-3D cells was found in only 1 out of 8 implantations (Table 1 and Fig. 5E and F). In addition, T3-2D cells implanted in the spleen also became engrafted in the lung, although at a lower frequency than in the liver (Fig. 5F). Given the apparently high metastatic and engraftment potential of T3-2D cells, we further tested transplantation into the stomach and abdominal cavity (Table 1). We found that T3-2D cells could become engrafted into the stomach and disseminate throughout the peritoneal cavity with a 100% frequency (Table 1 and Fig. 5G–J). Engraftment in the stomach was associated with metastasis to the liver, diaphragm and spleen, and peritoneal dissemination was associated with metastasis to liver, diaphragm, spleen, colon, pancreas and stomach. The above results show that the T3-2D cell line is a highly malignant gastric cancer cell line that metastasizes frequently to various organs.

### Normal immune system response and COX-2 signals increase the tumor engraftment and metastatic ability

When similar implantation experiments were performed using immunodeficient BALB/c-nu/nu mice, we unexpectedly observed a somewhat lower rate of tumor formation compared to the C57BL/6 immunocompetent mice (Table 2). In particular, we saw a significantly lower rate of tumor formation of the primary culture *p53*−/− Gan gastric epithelial cells and T3-3D cells subcutaneously injected into BALB/c-nu/nu mice compared to C57BL/6 mice. In addition, significantly lower rate of metastasis of T3-2D cells to the liver by transplantation into the spleen, a slightly lower rate of metastasis of T3-2D cells to the lung by splenic injection and metastasis to the liver and diaphragm by transplantation into the stomach was observed (Tables 1 and 2). These data show that the normal immune system response promotes tumor engraftment and metastasis.

**Table 2:** Tumor formation is suppressed in immunodeficient mice. Organoids derived from various genotypes were analyzed as in Table 1 using immunodeficient BALB/c-nu/nu mice. *Where there was a significant decrease in tumor formation frequency between C57BL/6 (Table 1) and BALB/c-nu/nu mice (Table 2). ^#^Where there was a decrease in frequency but this did not reach a statistically relevant difference.

Site	Cells	Donor mice	Recipient mice	Injections	Tumors	Liver	Lung	Diaphragm	Spleen	Colon & Pancreas	Stomach
Subcutis	**p53+/+ Gan**	2	3	4	0	0	0	0	0	0	0
	**p53+/− Gan**	4	6	6	1	0	0	0	0	0	0
	**p53−/− Gan**	4	4	6	3*	0	0	0	0	0	0
	**p53** **+/+** **Gan tumor**	4	4	6	0	0	0	0	0	0	0
	**p53** **+/−** **Gan tumor**	3	4	6	0	0	0	0	0	0	0
	**p53−/− Gan tumor**	2	5	6	0*	0	0	0	0	0	0
	**T3-3D**	1	6	6	2*	0	0	0	0	0	0
	**T3-2D**	1	4	5	5	0	2	0	0	0	0
Spleen	**T3-3D**	1	6	6	0	0	0	0	0	0	0
	**T3-2D**	1	8	8	8	3*	0^#^	0	0	0	0
Stomach	**T3-2D**	1	8	8	8	0^#^	0	0^#^	4	0	0

Next, the relationship between the inflammatory response, tumor engraftment and metastatic ability was analyzed. It has been reported that inhibition of the COX2 pathway results in suppression of tumor formation and inflammation of gastric tumors of Gan mice^25^. As shown in Fig. S4, administration of the anti-inflammatory COX-2 inhibitor, celecoxib, to Gan mice resulted in suppression of tumorigenesis and macrophage infiltration. We then analyzed the effect of the anti-inflammatory COX-2 inhibitor, celecoxib, to mice and analyzed its effect on implantation into the subcutis, spleen and peritoneal cavity. As shown in Table 3 and Fig. 5O, peritoneal dissemination was dramatically suppressed when celecoxib was administered. We also observed that celecoxib slightly suppressed subcutaneous tumor growth of T3-2D cells as shown in Fig. 5K and L. As shown in Table 3 and Fig. 5M and N, administration of celecoxib after cells had been transplanted into the spleen also slightly inhibited engraftment to the liver. We also analyzed the effect of celecoxib on macrophage infiltration within the engrafted tumors in liver. As shown in Fig. S5A and B, macrophage infiltration was decreased by celecoxib treatment to the levels similar to those in livers without tumors. We further analyzed the effect of celecoxib to T3-2D cells. As shown in Fig. S5C–F, celecoxib treatment resulted in dramatic decrease of *CXCL2* expression and N-cadherin expression, while total *CD44* expression was unchanged. The decrease of *CXCL2* and N-cadherin by celecoxib in T3-2D cells may have contributed to suppression of tumor engraftment and metastasis by celecoxib treatment in these cells. These results show that the normal immune system response and COX-2 signals promote tumor engraftment and metastasis of *p53*−/− Gan gastric epithelial cells and T3-3D and 2D cells.

**Table 3:** Tumorigenicity and metastatic potential of T3-2D cells are suppressed by an anti-inflammatory drug. T3-2D cells were analyzed for tumorigenicity and metastatic potential as in Table 1 using C57BL/6 mice with or without celecoxib (100 mg/kg/day), a COX-2 selective inhibitor, until the end of the study. Mice were observed for up to 1 month after subcutaneous implantation. After transplantation into the spleen and peritoneal cavity, mice were analyzed after 21 days. *Where there was a significant decrease in tumor formation frequency with or without celecoxib. ^#^Where there was a decrease in frequency but this did not reach a statistically relevant difference.

Site	Cells	Donor mice	Recipient mice	Injections	Tumors	Liver	Lung	Diaphragm	Spleen	Colon & Pancreas	Stomach
Subcutis	(**−**)	1	7	8	8	0	1	0	0	0	0
	(**+**)	1	4	6	6	0	0	0	0	0	0
Spleen	(**−**)	1	6	6	6	5^#^	0	1	0	0	0
	(**+**)	1	6	6	6	3^#^	0	1	0	0	0
I.P.	(**−**)	1	5	5	5*	2	1	5*	0	5*	0
	(**+**)	1	5	5	0*	0	0	0*	0	0*	0

### Loss of p53 function promotes tumorigenesis *in vivo*

Since Gan mice develop gastric tumors spontaneously, we next compared the gastric tumor tissues of *p53*+/+ Gan, *p53*+/− Gan, and *p53*−/− Gan mice. We first examined gene expression in the gastric tumors tissues that develop in *p53*+/+ Gan mice and asked if the p53 in these tumors was functional. *p53*+/+ Gan mice were irradiated with γ rays, and mRNA expression of three representative p53 target genes, *p21*, *PHLDA3* and *Mdm2*, were analyzed in the cancer tissues. As shown in Fig. S6A–C, irradiation efficiently induced expression these target genes, confirming that these cancers retain functional p53. We also analyzed *p53* gene copy number variation by PCR using PCR primers that distinguish the wild-type and the knock out alleles. As shown in Fig. S6D–G, both wild-type and p53 knock out alleles were detected in control tail tissue DNA of *p53*+/− mice, and similar intensities of both alleles were also detected using DNA from gastric tumor tissues of *p53*+/− Gan mice. These results show that functional p53 is present in the gastric tumor tissues of *p53*+/− Gan mice, as well as in gastric tumor tissues of *p53*+/+ Gan mice.

**Figure 6:**
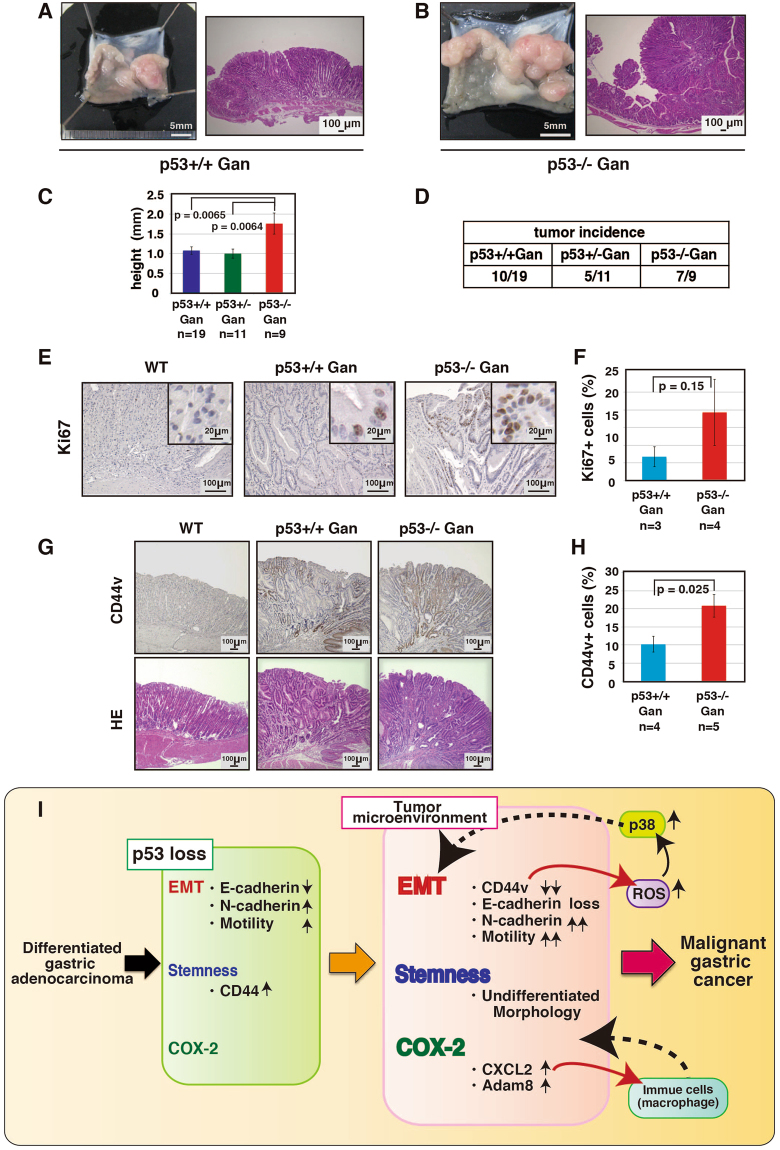
p53 loss of function promotes tumor growth. (**A**–**C**) Gastric cancer formation in 20–34 week-old *p53*+/+ Gan, *p53*+/− Gan and *p53*−/− Gan mice. Histological analysis was performed using H & E staining. Stomach height was measured as the height of the stomach from the length of the muscularis mucosa to the surface layer. The stomach height of *p53*−/− Gan mouse is higher than other mice. (**D**) Gastric cancer formation rate of *p53*+/+ Gan, *p53*+/− Gan and *p53*−/− Gan mice. When the length from the muscularis muscle plate to the surface layer was longer than 1200 μm, this was scored as a tumor. (**E**,**F**) Expression of Ki67 in stomach cancer of *p53*+/+ Gan, *p53*+/− Gan and *p53*−/− Gan mice. Ki67-positive cells were counted from immunohistochemically stained stomach cancer sections using TissueFAXS (TissueGnostics). (**G**,**H**) Expression of CD44v in stomach cancer from *p53*+/+ Gan, *p53*+/− Gan and *p53*−/− Gan mice. CD44v-positive cells were counted from immunohistochemically stained stomach cancer sections using TissueFAXS (TissueGnostics). (**I**) Development of malignant gastric cancer from gastric epithelial cells in a normal immune environment.

We next compared the height of the stomach, which is an indicator of tumor size, among age-matched *p53*+/+ Gan, *p53*+/− Gan, and *p53*−/− Gan mice. Stomach heights in the *p53*−/− Gan mouse were higher than the other mice (Figs. 6A–C). In addition, gastric tumor formation at 20-34 weeks of age was more frequent in the *p53*−/− Gan mice compared with other mice (Fig. 6D). We also have demonstrated that tumor development of *p53*−/− Gan mice depends on both C2mE and Wnt1 activity, and active β-catenin levels, an indicator of Wnt activity, was significantly elevated in both *p53*+/+ Gan and *p53*−/− Gan mice compared to wild-type mice (Fig. S7). Furthermore, the ratio of Ki67-positive to total cells in tumor tissues from *p53*−/− Gan mice tended to be higher than in the other mice strains (Fig. 6E and F). Next, we analyzed expression of CD44v in stomach tumors derived from *p53*+/+ Gan, *p53*+/− Gan, and *p53*−/− Gan mice. As shown in Fig. 6G and H, CD44v expression was higher in gastric tumor tissues from *p53*−/− Gan mouse. We also analyzed the tumor engraftment by gastric tumor cells from *p53*+/+ Gan, *p53*+/− Gan, and *p53*−/− Gan mice. As shown in Tables 1 and 2, only cells from the *p53*−/− Gan gastric tumors were tumorigenic. In addition, the tumorigenicity of *p53*−/− Gan gastric tumor cells were significantly lower when injected into BALB/c-nu/nu mice compared to C57BL/6 mice, suggesting that these cells require a normal immune system to form tumors. As far as we examined, there was no significant difference in tumorigenicity between gastric epithelial cells obtained from mice 7-10 weeks of age and gastric tumor cells. These results show that the loss of p53 function promotes the formation of gastric tumor *in vivo*.

## Discussion

Gan mice are transgenic mice that harbor activation of both the Wnt and PGE_2_ pathways and that spontaneously develop gastric tumors. To analyze the effect of loss of p53 function on gastric cancer formation and malignant transformation, we cultured gastric epithelial cells from *p53*−/− Gan mice *in vitro* and then transplanted these *in vivo*. We showed that gastric epithelial cells from *p53*-deficient mice and *p53*+/+ Gan mice were not tumorigenic, whereas gastric epithelial cells from *p53*−/− Gan mice were tumorigenic. These data show that gastric epithelial cells acquire tumorigenic ability as a result of the simultaneous activation of the Wnt pathway, the PGE_2_ pathway, and loss of p53 function.

Increased CD44 expression and induction of EMT in gastric epithelial cells from *p53*−/− Gan mouse were associated with the acquisition of tumorigenicity. It has been reported that p53 suppresses EMT and cell stemness by inducing transcription of the microRNA *miR-200c*^26^. We have shown that loss of p53 induces CD44 expression and EMT, accompanied by increased cellular motility and decreased E-cadherin expression in gastric epithelial cells prior to the development of gastric cancer. We also showed that this acquisition of tumorigenicity and EMT initiated by the loss of p53 function are further promoted by the tumor microenvironment *in vivo*, resulting in enhanced tumorigenicity, metastatic potential, and cell motility. In cancer cells, it has been reported that the splicing pattern of CD44 is altered as EMT progresses, marked by a shift in expression from CD44v to CD44s, the standard form of CD44^27^. We also observed that expression of CD44v is dramatically decreased in T3 cells that have undergone EMT, while total CD44 expression is slightly increased. It has been reported that CD44v suppresses p38 activation by decreasing ROS generation, while ROS triggers EMT through p38 activation^22^. In T3 cells, lower expression of CD44v may result in higher ROS generation and consequent induction of EMT by the ROS-p38 pathway. In normal cells, p38 activates p53, which suppresses EMT and cell stemness. However, in *p53*-deficient cells, ROS-p38 pathway activation-induced EMT goes unchecked. Gastric epithelial cells from *p53*−/− Gan mice acquire stem cell-like and mesenchymal properties due to the loss of p53 function, and p38 is further activated by the *in vivo* microenvironment, resulting in the induction of complete EMT and highly increased cell motility. Together these molecular changes may lead to the acquisition of a malignant gastric cancer phenotype (summarized in Fig. 6I).

It has been reported that p53 suppresses COX-2 expression^28,29^. It has also been reported that there is a correlation between the presence of p53 mutations and COX-2 expression in stomach cancer^30^. In Gan mice, the COX-2 pathway is already upregulated, but in the malignant T3 cells that developed within the *in vivo* microenvironment, the COX-2 pathway is abnormally activated to a greater extent. We have shown that this elevated COX-2 activity is required for stomach cancer formation and metastasis and is also essential for peritoneal dissemination. Activation of the COX-2 pathway was observed to increase the expression of CXCL-2 and other genes, which in turn may recruit immune cells to the tumor area to promote tumorigenesis (summarized in Fig. 6I).

The inflammatory response plays a major role in gastric cancer formation and malignancy, and we believe it is very important to study gastric cancer in the context of the normal immune system. However, such research has been difficult as there are no gastric cancer cells with a high metastatic potential to be used for allogeneic transplantation. Therefore, studies using gastric cancer cells are frequently performed by transplanting human gastric cancer cells into immunodeficient mice, which preludes analysis of the interactions between the immune system and gastric cancer cells. In this study, we report the establishment of an allogeneic transplantable malignant gastric cancer cell line, T3-2D. Engraftment of these cells into the stomach and peritoneal dissemination occurred at a frequency of 100%. Using this cell line, we showed that the normal immune system is necessary for the development of cancer and metastasis. With these T3-2D cells, we should now be able to analyze the involvement of immune cells in the development of malignant gastric cancer at the molecular level.

## Materials and Methods

### Mice Used in This Study

*K19-Wnt1/C2mE* transgenic (Gan) mice were described previously^7^. *p53*−/− Gan mice were generated by crossing the heterozygotes. Mouse experiments were performed in a specific pathogen-free environment at the National Cancer Center animal facility according to institutional guidelines, and all of the animal experiments were approved by the Committee for Ethics in Animal Experimentation at the National Cancer Center. C57BL/6 J and BALB/c-nu/nu mice were purchased from Jackson Laboratories (Charles River, Japan).

### Mouse Models

Compound *K19-Wnt1/C2mE* (Gan) transgenic mice were generated by crossing *K19-Wnt1* transgenic mice and *K19-C2mE* transgenic mice. Next, compound *p53*+/− *Wnt1* transgenic mice or *p53*+/− *C2mE* transgenic mice were generated by crossing *p53*−/− mice and *K19-C2mE* mice or *p53*−/− mice and *K19-Wnt1* mice. Finally, compound *p53*−/− *K19-Wnt1/C2mE* (Gan) mice were generated by crossing *p53*+/− *K19-C2mE* mice and *p5*3+/− *K19-Wnt1* mice or *p53*−/− *K19-C2mE* mice and *p53*+/− *K19-Wnt1* mice or *p53*+/− *K19-C2mE* transgenic mice and *p53*−/− *K19-Wnt1* mice.

Mice were treated with γ-rays (15 Gy) or not treated. Gastric tissues were dissected 6-7 hours post-irradiation, and mRNA expression of p53 target genes (*p21*, *PHLDA3* and *Mdm2*) were analyzed by real-time PCR.

### Organoid Culture Experiments

Organoid cultures of gastric epithelial cells and gastric tumors were performed as previously described^23^. For primary gastric epithelial cell cultures, glandular stomachs of mice were treated with 0.1% collagenase for 30 min followed by trypsin digestion, and cells were cultured in Matrigel (BD Biosciences, Franklin Lakes, NJ, USA & CORNING) with the primary culture medium containing 50ng/mL EGF (BD Biosciences) supplemented with 500 ng/mL R-spondin1 (R&D, Minneapolis, MN, USA), 2.5 ng/μl of Jagged1 (AnaSpec, Fremont, CA, USA), and 100 ng/mL of rMuNoggin (PeproTech, Rocky Hill, NJ, USA). The first primary cultured cells were cultured with CHIR99021 (2.5 μM; Stemgent, Cambridge, MA) at day 0^31^. The mean number of cystic structures >300 μm in diameter in Matrigel were counted at day 5~9.

### Transplantation experiments

Female or male C57BL/6 J and BALB/c-nu/nu mice at over 6 weeks of age were maintained in a specific pathogen-free environment at the National Cancer Center animal facility environment. Gastric epithelial cells of 7-10 week old mice, cells of gastric cancer formed in 33-48 week old mice, T1 cells, T2 cells, T3-3D cells and T3-2D cells were used for the analysis. All the cells except for T3-2D were seeded in Matrigel at a density of 1 × 10^5^ cells per 3-cm dish on day 0 and harvested on day 6-7 and used for the analysis. T3-2D cells were seeded at a density of 1.3 × 10^5^ cells per 10-cm dish and cultured for 2 days and used for the analysis. Implantation into the subcutis, spleen, stomach and peritoneal cavity were performed. The organoids were mechanically dissociated, and 1.3 × 10^5^ cells were injected together with Matrigel into mice. After subcutaneous implantation, the mice were observed until tumor growth ceased. After transplantation of cells into the spleen, stomach and peritoneal cavity, mice were observed for 21 days. For the cells that did not form tumors, the mice were observed for more than 1 month.

### Histological examination of organoids

The organoid cultures were prepared from gastric epithelial cells and T1-T3 cells, as previously described^23,31^. Cells were seeded at a density of 1.3 × 10^5^ cells per 3-cm dish on day 0 and analyzed on day 5. The cell suspension was recovered from Matrigel’s basement membrane matrix using Cell Recovery Solution (Corning). The cell suspension was hardened in jelly form with iPGell (GenoStaff) and fixed in 4% paraformaldehyde, paraffin embedded and sectioned at 5**-**μm thickness. The sections were stained with Haemotoxylin and Eosin.

### Immunofluorescence for organoids and T3-2D cells

The organoid cultures were prepared from gastric epithelial cells and T1-T3 cells, as previously described^23,31^. To obtain organoids, cells were seeded in Matrigel layered on cover glass at a density of 1 × 10^5^ cells per 12-well plate on day 0 and analyzed on day 5. T3-2D cells were seeded on cover glass at a density of 1 × 10^5^ cells per 12-well plate on day 0 and analyzed on day 1. Organoids or T3-2D cells were fixed in 4% paraformaldehyde. Primary antibody dilutions were 1:200 for CD44v (COSMO BIO), 1:500 for p-p38 (Cell signaling), 1:100 for E-cadherin (ECM), and 1:100 for N-cadherin (BD). Secondary antibodies (1:500 dilution for each) were Alexa Fluor 488 donkey anti-rat IgG for CD44v staining, Alexa Fluor 594 goat anti-rabbit IgG for p-p38 staining, and Alexa Fluor 488 goat anti-mouse IgG for E-cadherin or N-cadherin staining. DAPI (2.5 μg/ml) was used for nuclear staining. Fluorescent immunostaining was quantitatively analyzed using ImageJ.

### Histological examination, immunohistochemistry and immunofluorescence of tissues

Tissues cells were fixed in 4% paraformaldehyde, paraffin embedded and sectioned at 5**-**μm thickness. Immunohistochemistry (IHC) was performed basically according to the manufacturer**’**s instructions. In brief, after deparaffinization, tissues sections underwent antigen retrieval by autoclaving slides for 5 min in 10 mM citrate buffer (10 mM pH 6.0). Inactivation of endogenous peroxidase was carried out using 3% hydrogen peroxide water. Nonspecific interactions were blocked for 30 min using a 5% (vol/vol) goat serum solution. The Tissues were immunostained using antibody Ki67 (NOVUS), CD44 (BioLegend), CD44v (COSMO BIO) and F4/80 (BIO-Red). Primary antibody dilutions were 1:500 for Ki67, 1:200 for pan-CD44 and CD44v, and 1:100 for F4/80. For pan-CD44 and CD44v, Histofine Simple Stain (Nichirei Biosciences) was used for the secondary antibody reaction. For Ki67 staining, biotinylated anti-rabbit IgG antibody was used for the secondary antibody reaction, and the VECTASTAIN ABC KIT Standard (VECTOR LABORATORIES, INC.) was used for peroxidase staining. Finally, staining was visualized using diaminobenzidine-based peroxidase substrate (DAB). Hematoxylin staining was performed, followed by dehydration. Alexa Fluor 488 donkey anti-rat IgG (Life Technologies) was used for secondary fluorescent staining of F4/80, and DAPI (2.5 μg/ml) was used to stain nuclei. Secondary antibody dilutions were 1:200 for biotinylated anti-rabbit IgG, and 1:500 for Alexa Fluor 488 donkey anti-rat IgG. Ki67, CD44 and CD44v-positive cells were counted from immunohistochemically stained stomach cancer sections using TissueFAXS (TissueGnostics). Fluorescent immunostaining was quantitatively analyzed using ImageJ.

### Invasion and migration assays

Cell migration was determined using 24-well transwell plates with 8.0 μm pore sizes. Cell invasion was analyzed using the same transwell inserts coated with Matrigel. Matrigel-coated inserts were created by diluting Matrigel 1:200 with PBS, adding to the inserts (ThinCert; Grenier) and incubating for 3 hours at 37 °C. *p53*+/+ Gan, *p53*+/− Gan, *p53*−/− Gan and T3-3D cells (5 × 10^4^ cells) were seeded into the upper insert in serum-free medium, while medium with serum was placed in the lower well, and the plate was cultured for 24 hours. Next, the supernatant was discarded and the insert was fixed with PBS containing 4% paraformaldehyde. After that, Giemsa staining (Wako) was performed, and invading and migrating cells were counted using a microscope.

### Reverse Transcription and Real-Time PCR

Total RNA (500 ng) was used for reverse transcription. Reverse transcription was carried out using ReverTra Ace (TOYOBO) following the manufacturer’s instructions. Reverse-transcribed cDNAs were subjected to realtime PCR, which was performed with a CFX96 Touch Real-Time PCR System (Bio-Rad). For the detection of *CD44*, *Cdkn1a* (Mm.PT.58.5884610), *Mdm2* (Mm.PT.58.42166864), *CXCL2* (Mm.PT.58.10456839), *CD44* (Mm.PT.56a.33229033), a Prime Time Mini qPCR Assay from Integrated DNA Technologies was used. Custom-designed TaqMan Dual-Labeled Probes from Sigma were also used for the detection of *p53*, *PHLDA3*, *IER5*, *RPRM*, *PMAIP*, *PTP4A1*, *FUCA1*, *PLK2* and β*-actin*. SYBR Premix Ex Taq II (Takara) probe was used for detection of *Adam8* and *mPGES1*.

### Custom designed PCR primers for Real-time PCR

Mm_Trp53-P: CTATGGCTTCCACCTGGGCTTCC

Mm_Trp53-F: CTGTCATCTTTTGTCCCTTCTCAA

Mm_Trp53-R: TGAGGGGAGGAGAGTACGTG

Mm_Phlda3-P: CCCTCTCCCATCTCCGTGCCCAGAAG

Mm_Phlda3-F: GTCCTAAACCATGAGGCGTATCA

Mm_Phlda3-R: GTTGGTTTCACCTGTCTCTTCGAC

Mm_IER5-P: CCATCTCCTCGTCGGTGTGGTCCTCG

Mm_IER5-F: CGGCTCTACCCCTCTCAAGA

Mm_IER5-R: CCGAAGATGCTGATGAGGTTTG

Mm_RPRM-P: TTGGCATCTTCTTCCTTGGCTGCAACCTG

Mm_RPRM-F: GTGTGCTCTCGCTCACTGTG

Mm_RPRM-R: ACCACTGCCTCCACCTCTTTA

Mm_PMAIP1-P: AGGATGAGGAGCCCAAGCCCAACCC

Mm_PMAIP -F: GCCTGGGAAGTCGCAAAAGA

Mm_PMAIP -R: GCCGTAAATTCACTTTGTCTCCAA

Mm_PTP4A1-P: AGCTCCGGTGCTTGTTGCCCTAGC

Mm_PTP4A1-F: GGTTGCTGTATTGCTGTCCATTG

Mm_PTP4A1-R: CTCCACGCCGCTTTTGTCTTA

Mm_FUCA1-P: CCTCAAATCCCCAAAACGACCTCGGC

Mm_FUCA1-F: TACGCCACTTTCCTGTACTGG

Mm_FUCA1-R: TCTCCTTCTAGTCCTAGCATTGTTA

Mm_PLK2-P: CAGTCTGGCTGCCAAACCAAAGTCTCCC

Mm_PLK2-F: CAGGGATCTCAAGCTAGGGAAC

Mm_PLK2-R: GGGTTCCACATATTGTTCTCCCT

Mm_Actb-P: CACACCCGCCACCAGTTCGCCA

Mm_Actb-F: CGCGAGCACAGCTTCTTTG

Mm_Actb-R: CATGCCGGAGCCGTTGTC

Adam8-F: ACAGCAGCCTGCCAGCTAAGA

Adam8-R: TAAACAGGAACTGGGAGTGGTGAAC

Ptges (mPGES-1)-F: ACAGTGGTTTCAGCAGGGTGTC

Ptges (mPGES-1)-R: GTCCAGATTTGCAGCCAGGAG

### Drug administration

T3-2D cells were analyzed for tumorigenicity and metastatic potential as in Table 3 using C57BL/6 mice with or without celecoxib (100 mg/kg/day), a COX-2 selective inhibitor, until the end of the study. Celecoxib was administered as a 2% w/v solution in ethanol: PEG 400: water (22.2:66.6:11.2, v/v).

## Electronic supplementary material

Supplementary manuscript
