# Supplementary material for: Functional loss of p53 cooperates with the in vivo microenvironment to promote malignant progression of gastric cancers
Source: Sci Rep. 2018 Feb 2;8:2291. doi: 10.1038/s41598-018-20572-1 (PMC5797237; doi:10.1038/s41598-018-20572-1)

Functional loss of p53 cooperates with the *in vivo* microenvironment to promote malignant progression of gastric cancers

Junko Ohtsuka[1, 2], Hiroko Oshima[3], Issei Ezawa[1, 4], Ryo Abe[2], Masanobu Oshima[3], Rieko Ohki[1]

[1]Laboratory of Fundamental Oncology, National Cancer Center Research Institute, Tsukiji 5-1-1, Chuo-ku, Tokyo 104-0045, Japan

[2]Division of Immunobiology, Research Institute for Biomedical Sciences, Tokyo University of Science, 2669 Yamazaki, Noda City, Chiba 278-0022, Japan

[3]Division of Genetics, Cancer Research Institute, Kanazawa University, Kanazawa 920-1192, Japan

[4]Graduate School of Advanced Science and Engineering, Waseda University, 3-4-1 Okubo, Shinjuku-ku, Tokyo, 169-8555, Japan

Key words: p53, PGE_2, Wnt, gastric cancer, mouse model

*****Correspondence: rohki@ncc.go.jp

Supplementary

Fig. S1 Gastric epithelial cells of *p53*+/+ Gan mice have functional p53.

(A) Representative images of the organoids obtained from the indicated mice.

(B-K) Organoids were harvested, and expression of *p53* and the indicated p53 target genes in *p53*+/+ Gan, *p53*+/- Gan and *p53*-/- Gan organoids were analyzed by real-time PCR.

Fig. S2 Proliferative and self-renewal abilities of gastric epithelial cells of *p53*-/- Gan mouse depend on COX2 and Wnt1.

Gastric epithelial cells from *p53*+/- *C2mE*, *p53*+/- *Wnt1*, *p53*-/- *C2mE*, *p53*-/- *Wnt1* and *p53*-/- Gan mice at 21 weeks of age were cultured in Matrigel. After one passage, the cells were seeded at a density of 1.3 x 10^5 cells on day 0 and analyzed on day 7~9. Representative photographs of the primary cultured gastric epithelial cells in Matrigel are shown (A). The mean number of cystic structures >750 mm in diameter in Matrigel were counted and shown as a graph.

Fig. S3 In vivo microenvironment induced EMT, change in the splicing pattern of CD44, p38 activation and increase in COX2 pathway gene expression.

(A, D, F) Organoids were cultured in Matrigel on cover glass, and immunostaining of N-cadherin (A), CD44v (D) and p-p38 (F) were performed. Fluorescent immunostaining was quantitatively analyzed using ImageJ. Indicated numbers (n) of independent samples derived from different donor mice were analyzed.

(B, C) Invasion and migration assays were performed as in Fig. 3. The results presented are an average of 7~11 random microscopic fields. Indicated numbers (n) of independent samples derived from different donor mice were analyzed.

(E, G-I) Expression levels of *CD44*, *Adam8*, *CXCL2* and *mPGES-1* were analyzed by real-time PCR. Indicated numbers (n) of independent samples derived from different donor mice were analyzed.

Fig. S4 Celecoxib suppresses tumorigenesis and inflammation of gastric tumors of Gan mice.

40-44 week-old Gan mice were administrated with 1500 ppm celecoxib for 3 weeks.

(A) H&E staining of gastric tumors of Gan mice treated with or without celecoxib.
(B) Immunostaining for macrophage marker F4/80. Macrophage infiltration was decreased by celecoxib treatment in gastric tumors of Gan mice. Tissue sections were counter stained with E-cadherin and DAPI.

Fig. S5 Celecoxib treatment results in decreased macrophage infiltration into metastatic tumors derived from T3-2D cells and decreased expression of CXCL2 and N-cadherin in T3-2D cells.
(A, B) Immunostaining for macrophage marker F4/80. Macrophage infiltration was decreased by celecoxib treatment in metastatic tumors formed in liver from T3-2D cells. Fluorescent immunostaining was quantified using ImageJ.
(C, D) T3-2D cells were analyzed with or without celecoxib (50 μ M) for 24hs. Expression levels of *CXCL2* or *CD44* were analyzed by real-time PCR.
(E, F) T3-2D cells were cultured on cover glass, and immunostaining was performed using anti-N-cadherin antibody. N-cadherin positive cells were counted from 7 fields. Fluorescent immunostaining was quantitatively analyzed using ImageJ (F)

Fig. S6 Gastric tumors of *p53*+/+ Gan mice have functional p53.
(A-C) 8 month-old mice were treated with γ-rays (15 Gy) or not treated. Gastric tissues were dissected 6-7 hours post-irradiation, and mRNA expression of *p53* target genes (*p21, PHLDA3 and Mdm2)* were analyzed by real-time PCR.
(D-G) Copy number variation analysis of the DNA obtained from gastric cancers formed in 43-44 week-old *p53*+/+, *p53*+/- and *p53*-/- Gan mice and control tail DNA of *p53*+/+, *p53*+/- and *p53*-/- mice. PCR was performed in triplicate using PCR primers that distinguish the wild-type and the knock out allele (D, F). Intensities of each band were analyzed using ImageJ (E, G). There were no significant differences in the ratios of the wild-type and the knock out alleles between the samples.
PCR was performed using PCR primers shown below.
p53-F: ATC TCT GAC TAC ACA GAG AGG TGC
p53-R: TCC GTC ATG TGC TGT GAC TTC TTG

Fig. S7 Both Wnt1 and COX2 contribute to tumor development of *p53*-/- Gan mice.

(A, B) Activation of Wnt in the gastric tumors of *p53*+/+ Gan and *p53*-/- Gan mice (20-42 week-old). Whole cell lysates were prepared and analyzed by Western blotting. Antibodies against unphosphorylated active-β-catenin (Millipore) and β-actin (Sigma) were used. Signal intensities of active β-catenin were quantified and normalized to β-actin.

(C-E) Gastric cancer is not formed in 15-28 week-old *p53*-/- *Wnt1* and *p53*-/- *C2mE* mice.

Fig. S8 For Fig. 1B, the numbers of cystic structures from each mice are shown.

Fig. S9 Full gel images of Figs. S6D and S6F.

Fig. S10 Full blot images of Fig. S7A.

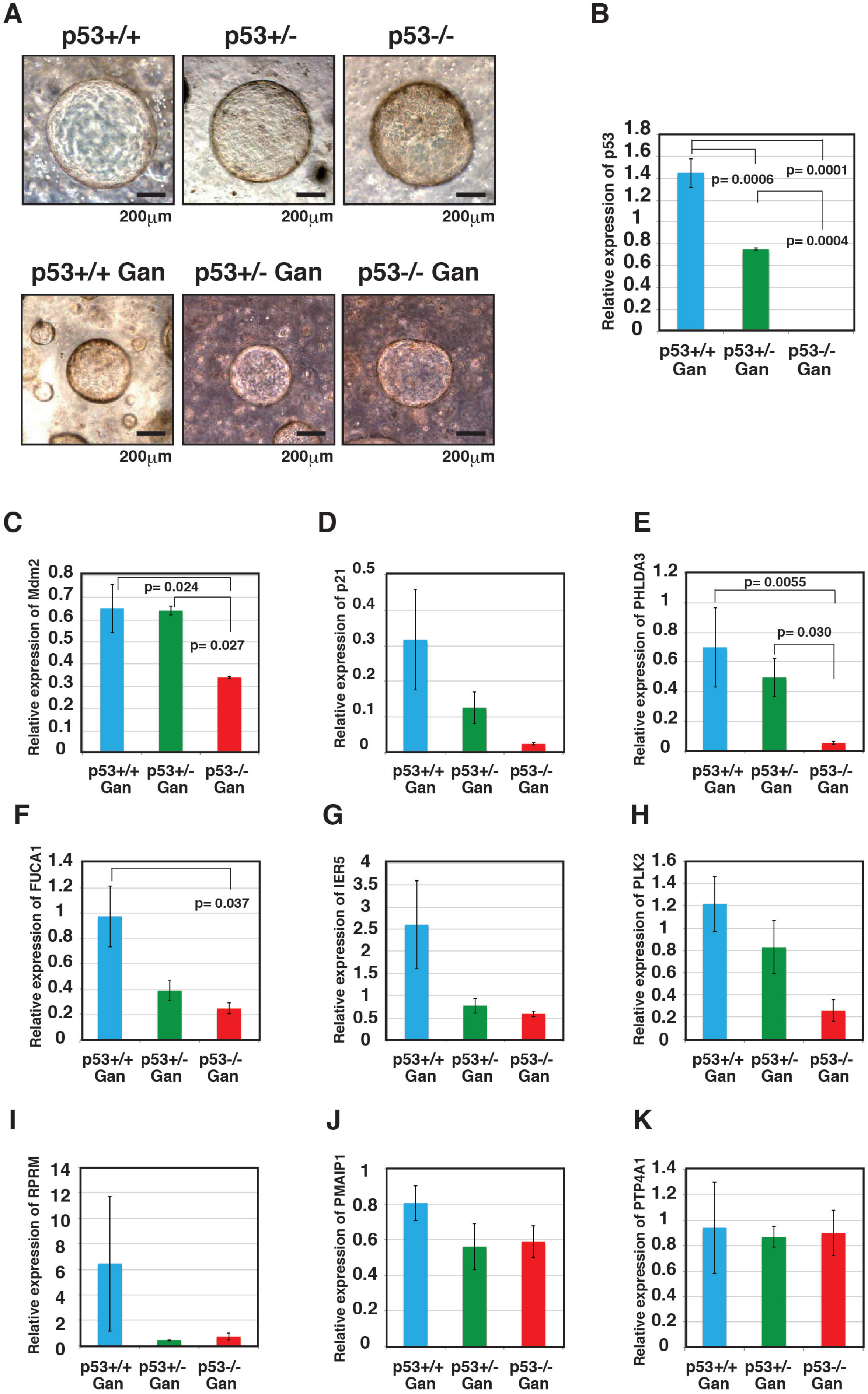

A
p53+/+
p53+/-
p53-/-
200μm
200μm
200μm
p53+/+ Gan
p53+/- Gan
p53-/- Gan
200μm
200μm
200μm
B
Relative expression of p53
p= 0.0006
p= 0.0001
p= 0.0004
p53+/+ Gan
p53+/- Gan
p53-/- Gan
C
Relative expression of Mdm2
p= 0.024
p= 0.027
D
Relative expression of p21
E
Relative expression of PHLDA3
p= 0.0055
p= 0.030
F
Relative expression of FUCA1
p= 0.037
G
Relative expression of IER5
H
Relative expression of PLK2
I
Relative expression of RPRM
J
Relative expression of PMAIP1
K
Relative expression of PTP4A1

A

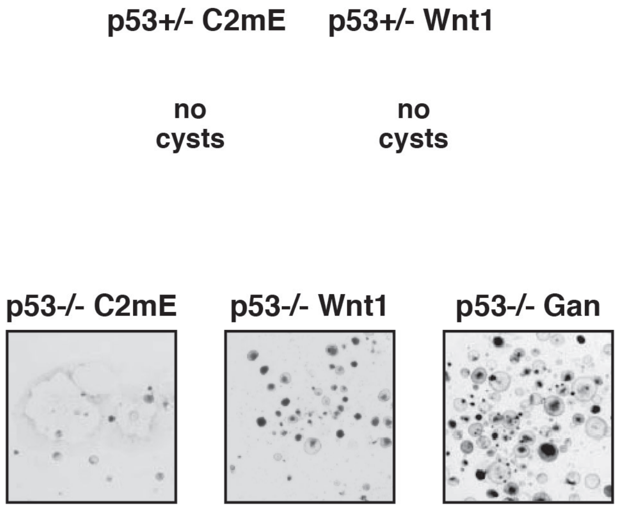
p53+/- C2mE
p53+/- Wnt1
no cysts
no cysts
p53-/- C2mE
p53-/- Wnt1
p53-/- Gan

B

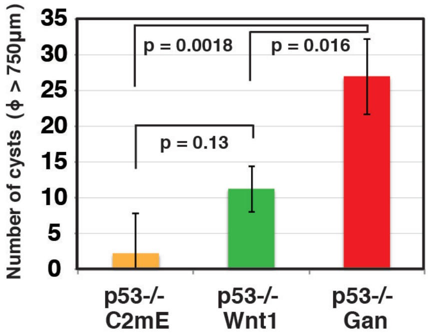
Number of cysts (ϕ > 750μm)
35
30
25
20
15
10
5
0
p = 0.0018
p = 0.016
p = 0.13
p53-/- C2mE
p53-/- Wnt1
p53-/- Gan

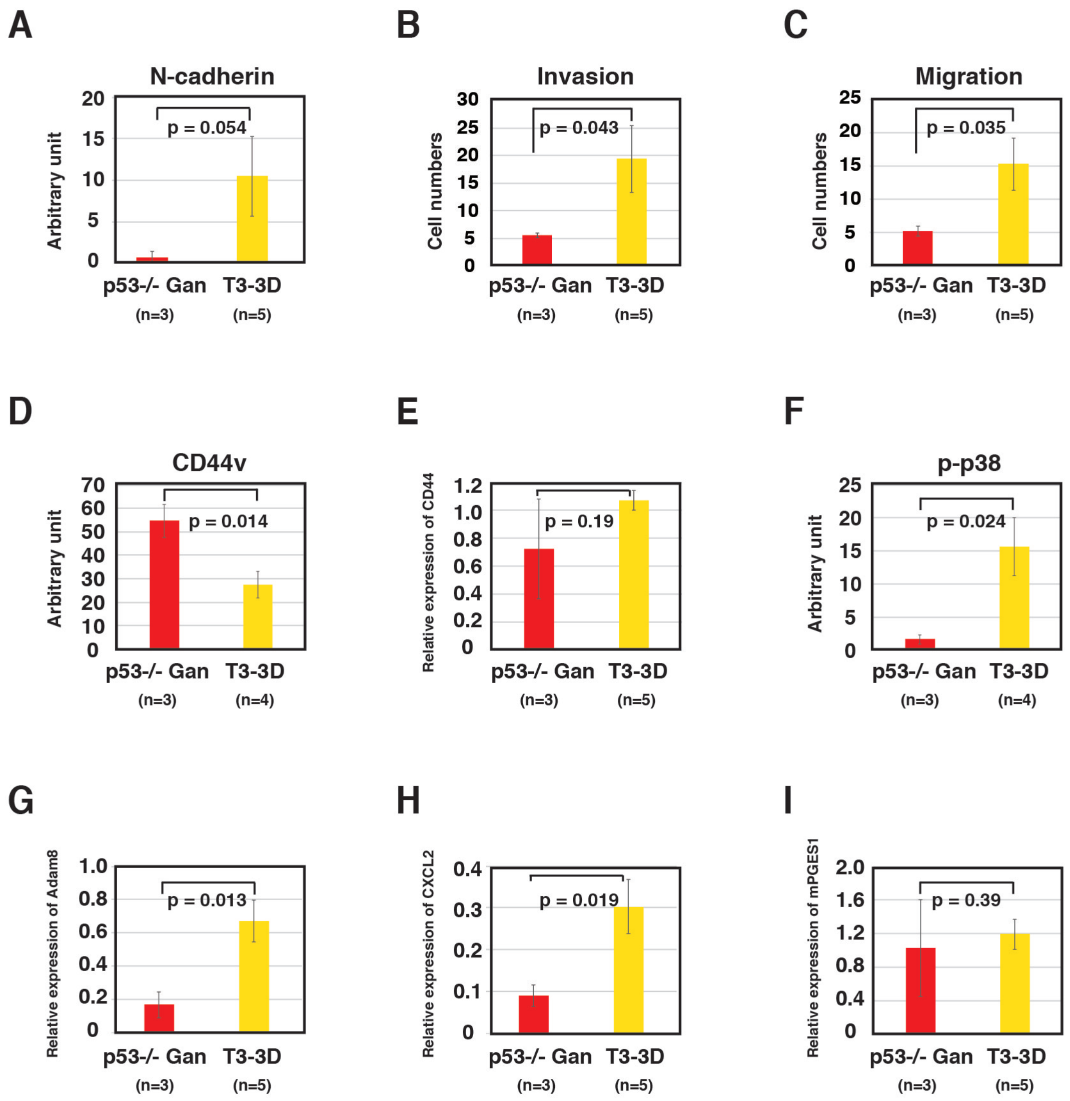
A
N-cadherin
Arbitrary unit
p = 0.054
p53-/- Gan
T3-3D
(n=3)
(n=5)
B
Invasion
Cell numbers
p = 0.043
p53-/- Gan
T3-3D
(n=3)
(n=5)
C
Migration
Cell numbers
p = 0.035
p53-/- Gan
T3-3D
(n=3)
(n=5)
D
CD44v
Arbitrary unit
p = 0.014
p53-/- Gan
T3-3D
(n=3)
(n=4)
E
Relative expression of CD44
p = 0.19
p53-/- Gan
T3-3D
(n=3)
(n=5)
F
p-p38
Arbitrary unit
p = 0.024
p53-/- Gan
T3-3D
(n=3)
(n=4)
G
Relative expression of Adam8
p = 0.013
p53-/- Gan
T3-3D
(n=3)
(n=5)
H
Relative expression of CXCL2
p = 0.019
p53-/- Gan
T3-3D
(n=3)
(n=5)
I
Relative expression of mPGES1
p = 0.39
p53-/- Gan
T3-3D
(n=3)
(n=5)

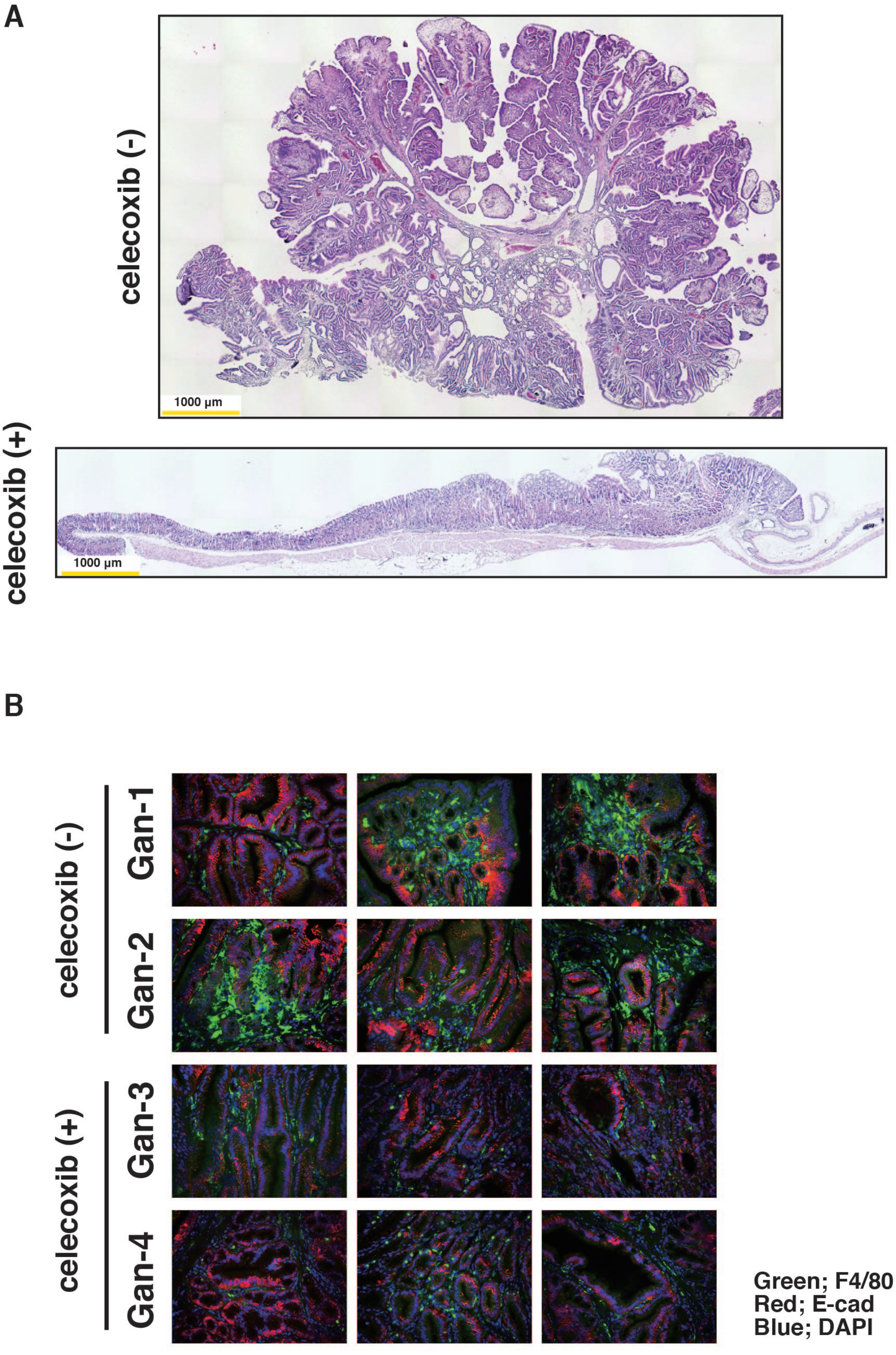
A
celecoxib (-)
1000 µm
celecoxib (+)
1000 µm
B
celecoxib (-)
Gan-1
Gan-2
celecoxib (+)
Gan-3
Gan-4
Green; F4/80
Red; E-cad
Blue; DAPI

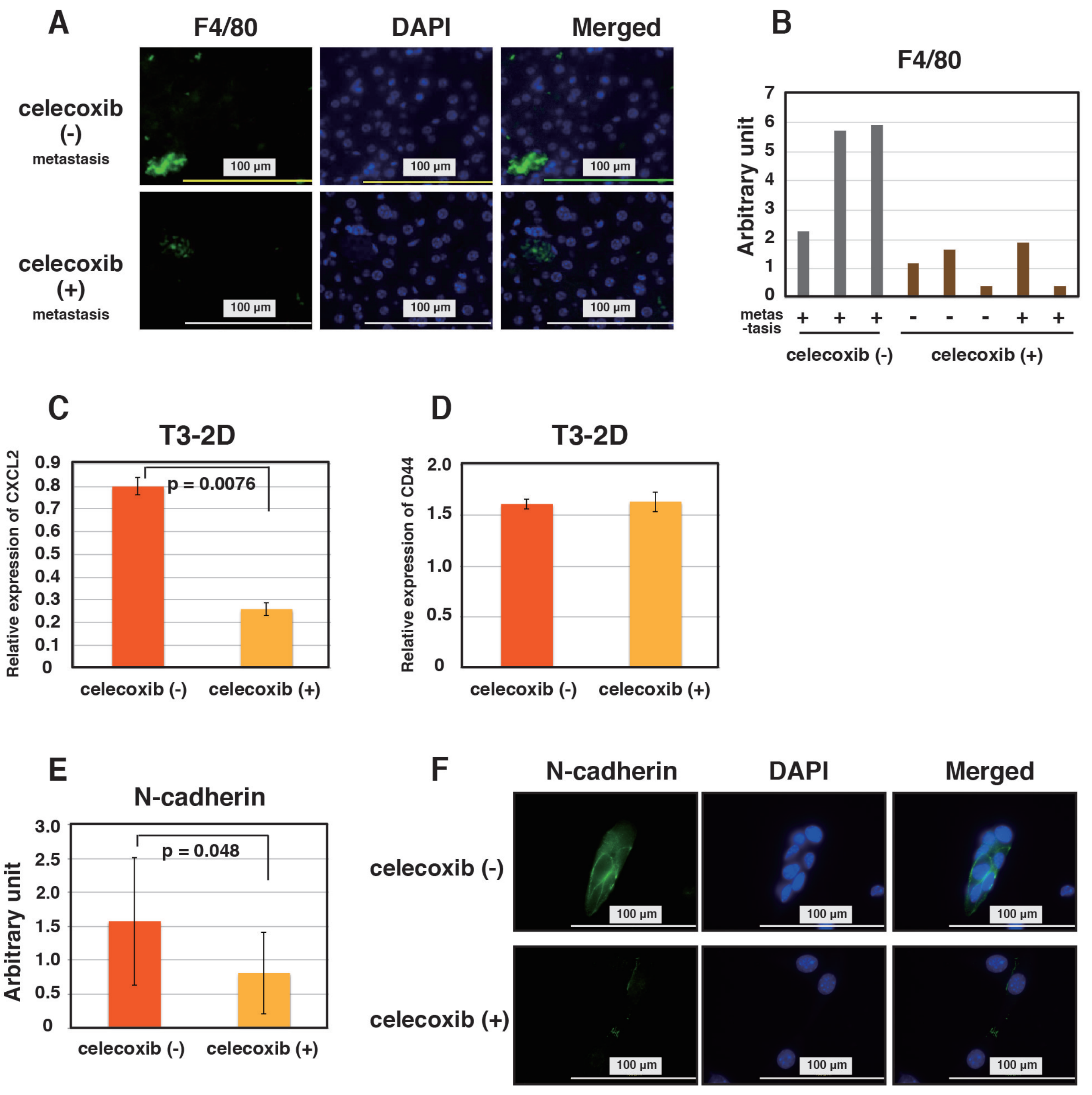
A
F4/80
DAPI
Merged
celecoxib (-) metastasis
celecoxib (+) metastasis
100 μm
B
F4/80
Arbitrary unit
7
6
5
4
3
2
1
0
metas -tasis
+ + + - - - + +
celecoxib (-)
celecoxib (+)
C
T3-2D
Relative expression of CXCL2
0.9
0.8
0.7
0.6
0.5
0.4
0.3
0.2
0.1
0
p = 0.0076
celecoxib (-)
celecoxib (+)
D
T3-2D
Relative expression of CD44
2.0
1.5
1.0
0.5
0
celecoxib (-)
celecoxib (+)
E
N-cadherin
Arbitrary unit
3.0
2.5
2.0
1.5
1.0
0.5
0
p = 0.048
celecoxib (-)
celecoxib (+)
F
N-cadherin
DAPI
Merged
celecoxib (-)
celecoxib (+)
100 μm

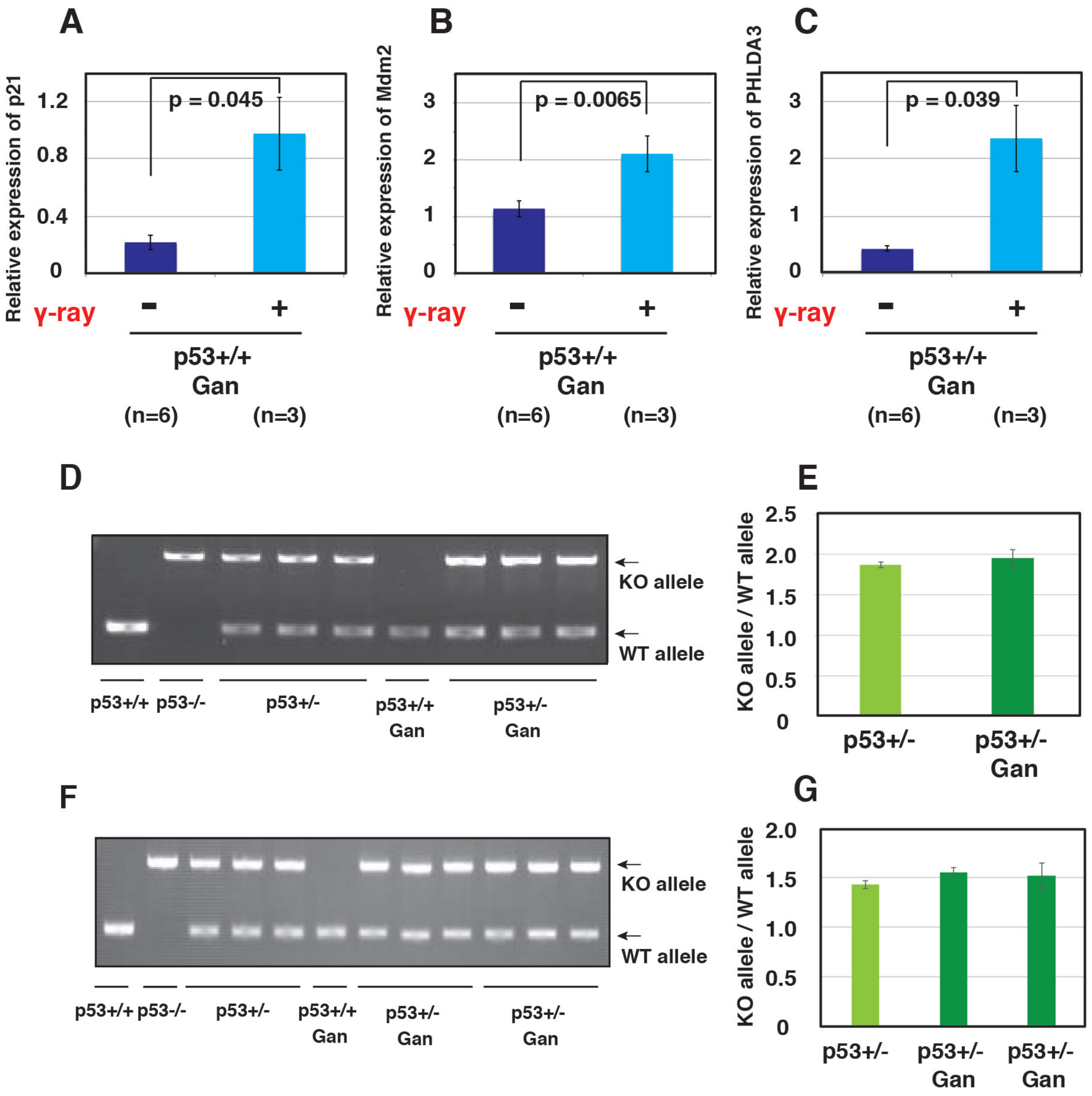

A
B
C
Relative expression of p21
Relative expression of Mdm2
Relative expression of PHLDA3
p = 0.045
p = 0.0065
p = 0.039
γ-ray
-
+
p53+/+
Gan
(n=6)
(n=3)
D
KO allele
WT allele
p53+/+ p53-/-
p53+/-
p53+/+
Gan
p53+/-
Gan
E
KO allele / WT allele
p53+/-
p53+/-
Gan
F
G

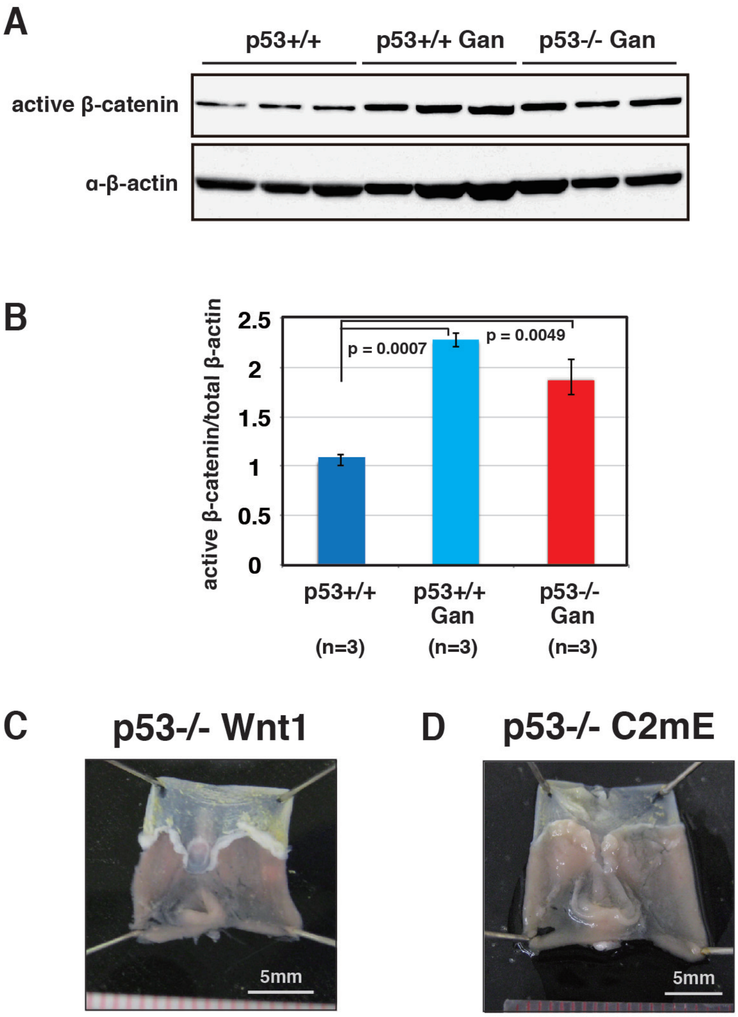

E

tumor incidence	
p53-/- Wnt	p53-/- C2mE
0/5	0/5

	p53+/+	p53+/-	p53-/-	p53+/+Gan	p53+/-Gan	p53-/-Gan
1	7	15	17	26	29	74
2	2	7	12	56	64	68
3	7	10	25	6	11	27

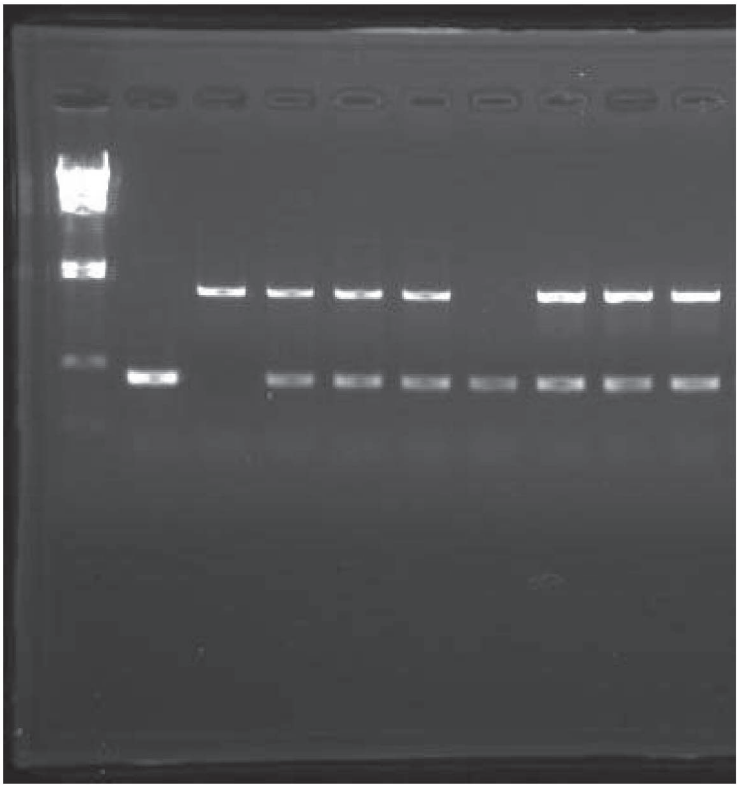

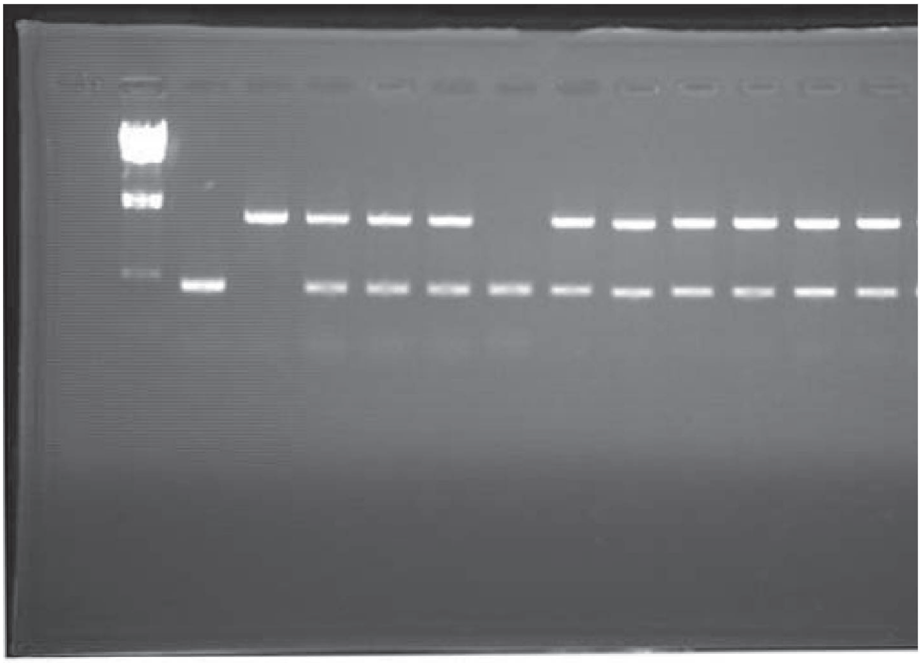

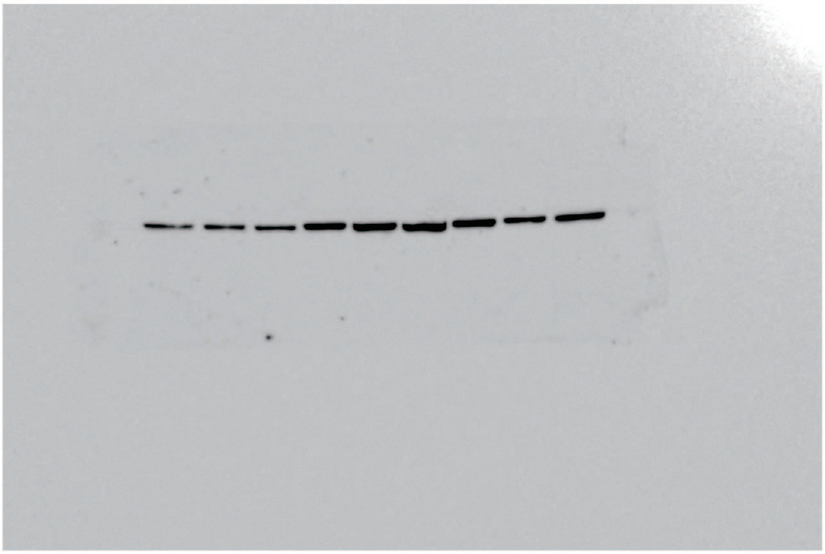

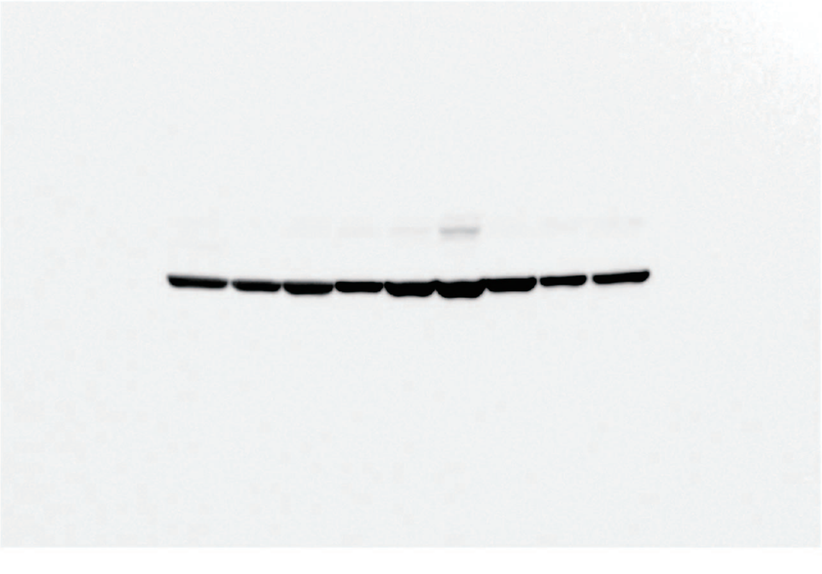

Supplement: Supplementary file 1 — Supplementary manuscript [file 41598_2018_20572_MOESM1_ESM.pdf]
